# Emerging Environmental Toxicants Undermine Reproductive Success in Aquatic Animals: A Narrative Review

**DOI:** 10.3390/jox16040137

**Published:** 2026-07-27

**Authors:** Yuchen Du, Hua Chang, Qiuyue Wang, Yaqin Liang, Liqian Shang, Chun Yang, Ling Li, Xun Xiang

**Affiliations:** 1 College of Animal Science and Technology, Yunnan Agricultural University, Kunming 650201, China; 2 College of Veterinary Medicine, Yunnan Agricultural University, Kunming 650201, China

**Keywords:** microplastics, nanoplastics, PFAS, endocrine-disrupting chemicals, reproduction, reproductive toxicity, aquatic ecotoxicology, mixture toxicity, bioaccumulation, transgenerational effects

## Abstract

Aquatic animals are increasingly exposed to complex mixtures of emerging environmental toxicants, including microplastics, nanoplastics, per- and polyfluoroalkyl substances (PFAS), and endocrine-disrupting chemicals (EDCs). This raises serious concerns for reproductive health, offspring survival rates, and long-term population stability. This narrative review synthesizes the available peer-reviewed evidence on the reproductive and developmental effects of these contaminant groups in aquatic animals. Growing evidence indicates that these contaminants adversely affect reproductive processes, impair offspring development, and reduce survival and fitness across a wide range of aquatic species. Their effects are mediated through interconnected pathways involving oxidative stress, endocrine disruption, inflammation, mitochondrial dysfunction, and epigenetic alterations, ultimately leading to reproductive and developmental abnormalities. These changes may weaken wild fish populations, reduce aquaculture productivity, and compromise aquatic biodiversity. However, interpretation of the available evidence is constrained by substantial heterogeneity in species, contaminant properties and concentrations, exposure durations, and reproductive endpoints, together with the predominance of laboratory-based single-contaminant studies. This review integrates current knowledge on the reproductive and developmental impacts of emerging contaminants by connecting mechanistic toxicity pathways with population-level and ecosystem consequences. Overall, this review emphasizes that protecting aquatic reproductive health requires an integrated framework linking contaminant monitoring, mechanistic biomarkers, reproductive performance, and ecosystem-level risk assessment.

## 1. Introduction

Reproduction in aquatic organisms becomes highly vulnerable due to environmental pollution because their reproduction is controlled through hormone signaling, gonadogenesis, gametogenesis, fertilization, embryonic development, and survival of offspring [1]. The term emerging contaminants refers to a diverse range of new types of anthropogenic chemicals or particles that have been identified, are increasingly being detected, but which are neither routinely monitored nor comprehensively regulated, despite growing evidence of ecological and biological effects [2]. Emerging contaminants in aquatic systems are mostly of four types, namely micro- and nanoplastics [3], per- and polyfluoroalkyl substances (PFAS) [4,5], and endocrine-disrupting chemicals (EDCs), like bisphenols [3], phthalates [6], and some pesticides which are known to have endocrine effects. This is of particular importance owing to their persistence or constant generation, bio-accumulative properties, and ability to work with other stressors, which makes them more likely to cause reproduction and development problems in aquatic organisms [5,7,8]. However, the magnitude of exposure is considerable because global emissions of primary and usage-related secondary microplastics were calculated to be about 2.7 million tons in 2020 and are forecasted to rise to 4.1 million tons by 2040 in the baseline scenario [9]. Additionally, PFAS are considered relevant to the aquatic environment around the globe since these compounds are resistant, ubiquitous in the environment and biological tissues, and some of them may bioaccumulate in aquatic animals [4].

Microplastics and nanoplastics are included among emerging contaminants because their occurrence in aquatic systems, small particle size, capacity to interact with biological barriers, and potential to carry or modify the behavior of other contaminants may increase their biological availability and toxicity [8]. Micro- and nanoplastics can impair reproductive function through particle accumulation, oxidative stress, inflammation, mitochondrial dysfunction, endocrine disruption, and altered expression of steroidogenesis-related genes in fish gonads [8,10]. Since micro- and nanoplastics can pass through biological barriers and accumulate within the fish gonad tissue, it is possible that reproductive disorders associated with micro- and nanoplastics might be not only direct but transgenerational [8]. For instance, female zebrafish exposed to polystyrene microplastics showed a reduced gonadosomatic index and fecundity, increased reactive oxygen species levels, hormonal imbalance, and altered expression of steroidogenesis-related genes [10]. In addition to their direct effects, microplastics may modify the uptake, distribution, and toxicity of co-occurring contaminants in aquatic organisms [11]. Polystyrene microplastics have been shown to increase microcystin-LR accumulation in zebrafish gonads, aggravate gonadal damage, disrupt sex-hormone balance, and alter gene expression associated with the hypothalamic–pituitary–gonadal axis [11].

PFAS represent a major group of emerging contaminants because many of these compounds are highly persistent in the environment, while their mobility and bioaccumulation potential vary substantially with carbon-chain length and functional group. Long-chain PFAS generally show greater bioaccumulation and trophic transfer in aquatic organisms, whereas short-chain alternatives, including PFBS, are comparatively more mobile and may remain difficult to remove from aquatic systems [4,12]. Current evidence indicates that PFAS-induced reproductive toxicity mainly involves gonadal damage, disruption of sex-hormone regulation and adverse effects on offspring development [5]. Developmental exposure studies in zebrafish have shown that environmentally relevant exposure to perfluorooctane sulfonate (PFOS) can reduce adult egg production, while both PFOS and perfluorobutane sulfonate/perfluorobutane sulfonic acid (PFBS) can alter growth, organ development, anxiety-like behavior and liver lipid profiles [13]. These findings underscore the need for PFAS risk assessment frameworks that account for compound-specific properties, exposure conditions, and life-stage-dependent reproductive susceptibility [5,13].

Endocrine-disrupting chemicals (EDCs) are another important class of emerging contaminants because they can mimic, block, or otherwise modify endogenous hormonal-signaling pathways that regulate sex differentiation, gamete maturation, spawning, embryonic development, and offspring fitness [3,14]. EDCs, including bisphenol A (BPA), BPA analogs, phthalates and pesticides, further increase reproductive risk because they can mimic, block or modify endogenous hormonal signals involved in sex differentiation, gamete maturation, spawning and embryonic development [3]. Bisphenols are particularly relevant in fish because they can affect estrogenic, androgenic and thyroid-related pathways that regulate reproductive physiology and developmental competence [3]. Combined exposure is especially important in aquatic systems, as polyethylene microplastics and BPA produced stronger endocrine and cellular disruption than single exposures in zebrafish, including altered hypothalamic–pituitary–gonadal axis gene expression [7]. Although several reviews have examined the effects of individual contaminant groups, such as microplastics, PFAS, or endocrine-disrupting chemicals, most studies have focused on specific pollutants, species, or isolated toxicological endpoints. Consequently, a comprehensive understanding of how these major classes of emerging contaminants collectively affect reproductive performance across different aquatic taxa remains limited. In addition, the links between molecular and physiological mechanisms of toxicity, reproductive outcomes, offspring development, and broader ecological consequences have not been systematically integrated. It is crucial to fill these gaps due to increasing exposure of aquatic organisms to multiple pollutants, leading to additive or synergistic effects on reproductive physiology. This is why this narrative review aims at discussing the existing evidence on microplastics’, nanoplastics’, PFAS’, and EDCs’ detrimental effects on the reproductive physiology of aquatic organisms, paying attention to mechanisms of exposure, endocrine disruption, gonad impairment, gametes quality, embryo development, fecundity, hatchability, fitness of the offsprings, and knowledge gaps in the field of reproductive toxicology.

## 2. Data Collection and Selection Criteria

This narrative review was conducted using a structured literature search approach to synthesize current knowledge on the reproductive and developmental effects of emerging environmental contaminants in aquatic animals. Relevant peer-reviewed studies were identified through comprehensive searches of major scientific databases, including Web of Science, Scopus, PubMed, ScienceDirect, Google Scholar, and ResearchGate. Literature searches were performed using combinations of keywords such as “microplastics,” “nanoplastics,” “per- and polyfluoroalkyl substances (PFAS),” “endocrine-disrupting chemicals,” “bisphenol A,” “phthalates,” “pesticides,” “aquatic animals,” “fish reproduction,” “reproductive toxicity,” “gametogenesis,” “embryo development,” “larval development,” “oxidative stress,” “endocrine disruption,” “mitochondrial dysfunction,” “epigenetic alteration,” “mixture toxicity,” “bioaccumulation,” and “transgenerational effects.” Boolean operators (AND, OR) were applied to optimize search specificity and coverage. Peer-reviewed articles published in English up to 2026 were considered. Priority was given to recent experimental studies, field investigations, mechanistic toxicological studies, and comprehensive review articles that evaluated the reproductive and developmental effects of microplastics, nanoplastics, PFAS, endocrine-disrupting chemicals, or their mixtures in aquatic organisms. Relevant studies involving aquatic invertebrates, including crustaceans, mollusks, oysters, scallops, and copepods, were also included when they provided mechanistic or ecological insights into contaminant-induced reproductive toxicity. The review excluded non-peer-reviewed publications, conference abstracts lacking complete experimental data, editorials, opinion articles, non-English publications, and studies that did not evaluate reproductive, developmental, endocrine, or mechanistic toxicological outcomes relevant to aquatic organisms. Preference was also given to studies reporting environmentally relevant exposure concentrations, well-defined reproductive endpoints, and mechanistic biomarkers that contribute to understanding the links between contaminant exposure, reproductive impairment, offspring development, and population-level ecological consequences.

## 3. Sources, Occurrence and Exposure Pathways of Emerging Contaminants in Aquatic Environments

### 3.1. Major Sources of Microplastics, Nanoplastics, PFAS and EDCs in Aquatic Systems

The aquatic ecosystem faces continuous inputs of various multiple point source pollutants including microplastics, nanoplastics, PFAS, and endocrine-disrupting substances with independent origins that overlap each other [2]. Microplastics may get into the environment through plastic waste breakdown, wastewater, stormwater, synthetic textile fibers, abrasion from tires, industrial pellets, fishing gear, and plastics associated with aquaculture [15]. Synthetic textile is responsible for 35% of the total global emissions of microplastics, followed by abrasion from tires contributing 28%, and urban dust accounting for 24% [16]. Furthermore, there is a large quantity of fiber that gets released when synthetic clothes get washed. According to De Falco et al. [17], there is a release of 640,000–1,500,000 microfibers during one wash cycle based on the type of the fabric. Nevertheless, the importance of these sources from the point of view of risk assessment is determined not only by the quantities of emissions. Wastewater and urban runoff have always been regarded as the most important pathways since microplastics travel continuously into water bodies from these pathways [18].

The other category of concern includes synthetic fiber textiles because they make up the largest amount of microplastic pollutants in both freshwater and marine waters and can be easily ingested by the organisms living in these environments. On the contrary, there is a possibility that the tire wear particles (TWPs) might pose a disproportionate amount of ecological risks because of the presence of additives, metals, and organic pollutants in them which apart from being plastic particles can prove to be toxic as well. This suggests that besides emissions, ecological risk assessment needs to consider other issues such as the characteristics of the source [19].

The contribution from TWPs is especially noteworthy. In a recent estimate, it was found that about 1.3 million metric tons of TWPs are produced every year in Europe, with Germany, France, and Italy contributing more than 100,000 tons each per year [20]. Wastewater treatment plants are simultaneously interceptors and sources, since membrane bioreactors, together with tertiary filters, allow removal efficiencies greater than 99%; however, the amount of treated water is so huge that the average microplastic load in effluent remains relatively high—between 0.2 and 8.3 mg per capita per day [21,22]. Similar issues have been found in other areas as well. Road traffic in North America is said to be one of the biggest sources of microplastic discharge into water bodies, and fast-growing numbers of vehicles in Asian countries, like China and India, will further lead to an increased production of TWP and its release into the environment [23]. These regional analyses suggest that the problem of TWP pollution is a worldwide one that is intimately tied with transportation intensity, population density, and urbanization.

Advanced systems for wastewater treatment achieve efficiencies of up to 99.85% regarding the removal of microplastics in the dry season; nonetheless, the rainy season may affect the efficiency level [24]. Despite the high removal efficiencies reported for advanced wastewater treatment technologies, contaminants continue to enter aquatic systems through several pathways. treatment performance can vary depending on operational conditions, infrastructure age, maintenance practices, hydraulic loading, and the size and physicochemical properties of contaminant particles. Smaller particles, particularly nanoplastics, may be more difficult to capture and therefore remain in treated effluents. Furthermore, many contaminants removed from wastewater are transferred to sewage sludge rather than completely eliminated [25].

From these larger particles, nanoplastics are generated through multiple converging mechanisms. Conventionally, they arise from the further weathering, abrasion, and degradation of larger plastic debris, including tire particles. However, recent research demonstrates that nanoplastics can also be released directly from macroplastics through chain scission and oxidation-driven embrittlement, bypassing the microplastic stage entirely [26]. Landfills are key secondary sources of generation of micro- and nanoplastics, with approximately half of the plastic waste going there. Total plastic waste concentrations in landfills vary between 85 and 414 mg per g, while microplastics, fine microplastics, and nanoplastics concentrations in landfills amount to 2–69, 0.5–36.8, and 0.04–1.9 mg per g of plastic waste, respectively. Modeling shows that half of the landfilled plastics will fragment into micro- and nanoplastics within 83–189 years, with the highly complex conditions of landfills resulting in the exponential fragmentation of plastic waste over time [27].

Turning to persistent synthetic chemicals, PFAS contamination originates from industrial discharge, wastewater treatment plants, landfill leachate, firefighting foams (aqueous film-forming foams at airports and military bases), textile and paper products, and PFAS-containing consumer materials [28]. In a recent global assessment of PFAS contamination in industrial effluent, data from 205 industrial facilities located in Asia, Europe, and North America revealed specific PFAS signatures among the fluorochemicals, electronics, electroplating, and textiles sectors [28]. Moreover, municipal and industrial WWTPs exhibit low PFAS removal efficiencies. Systematic literature analysis of 65 papers in 23 countries proved that, although innovative technologies like reverse osmosis and granular activated carbon allow removal rates higher than 90%, they produce secondary streams with high concentrations of PFAS that need further management and fail to destroy these compounds [29].

Most importantly, however, endocrine-disrupting chemicals such as bisphenols, phthalates, pesticides, pharmaceuticals, and synthetic hormones get into aquatic ecosystems via municipal wastewaters, agricultural runoff, industrial wastes, landfill leachate, and from plastics [14]. Source mechanisms vary depending on the nature of the compounds: pesticides generally emanate from upstream agricultural operations where they become diluted downstream, but pharmaceuticals and industrial chemicals emanate from sewage disposal systems and industries. In agricultural areas, the use of manure fertilizers with remnants of estrogen hormones leads to migration of the hormones to the groundwater through fertilizer application and wastewater irrigation [30]. The freshwater resources are still very susceptible to pollution through runoff from farms, waste from animals, and industrial waste, and there have been instances where a combination of pesticides like atrazine and metolachlor in agricultural watersheds has caused hormonal imbalance among aquatic animals [31]. Collectively, these interrelated yet varied mechanisms ensure that there is a constant input of mixed contaminants into water bodies. It follows, therefore, that aquatic organisms are not exposed to just one kind of contaminant at any one time, but rather to a combination of microplastics, nanoplastics, PFAS and EDCs. The major sources and pathways are summarized in Table 1.

### 3.2. Environmental Distribution in Water, Sediment and Suspended Particles

Upon entry into aquatic environments, EDCs do not have an even distribution in these systems but are separated into water, suspended particulate materials, and sediment based on their physical and chemical characteristics. Microplastics can either be suspended in the water column, deposited in sediments, or alternatively resuspended due to hydrodynamic disturbance. Factors such as aggregation, sedimentation, burial, and resuspension regulate their transport between the different environments [37]. Gao et al. [38] showed that reservoir systems can act as important sinks for microplastics, with dams influencing the transfer of particles from water to sediment and altering their spatial distribution.

PFAS distribution also varies between water, suspended particles and sediments, depending on chain length, functional group, salinity, organic matter and hydrological conditions. In coastal plain water networks, Wang et al. [39] observed different PFAS distribution patterns between surface water and sediments and suggested that sediments can be an important management target for PFAS pollution in such systems. The same applies to EDCs, which have been observed to exhibit compartment-specific behavior; as noted by Chen et al. [40], certain EDCs present in the coastal aquatic germplasm reserves have been found in the water dissolved, suspended particulates and surface sediments, where their partitioning depends on seasons as well as their characteristics. In general, sediments and suspended particulates cannot just be viewed as passive storage sites but also possible secondary sources.

### 3.3. Biological Exposure Routes in Fish and Other Aquatic Animals

Aquatic animals are exposed to emerging contaminants through direct contact with contaminated water, ingestion of suspended particles or sediment, trophic transfer through prey and uptake across epithelial surfaces such as the gills, skin and digestive tract. For microplastics, fish can be exposed through direct oral ingestion from the water column or contaminated sediments and indirectly through trophic transfer via contaminated prey. Particles suspended in the water may be retained on the surface of gills during respiration [41]. Embryos and larvae can be particularly sensitive due to incomplete organ formation and the need for a lot of energy for growth and development, which means that their capability for detoxification is lower compared to the capability of adults [42]. The PFAS exposure occurs through both water and food. The water exposure is through the absorption of water by the gills and surface area of the fish in the aquatic environment, while the food exposure occurs due to the contamination of the food in aquatic ecosystems. [43]. Regarding EDCs, the main routes through which they interact with the organism include gill interaction, ingestion through contaminated food or sediments, and dermal interactions [14]. These multiple exposure routes mean that aquatic organisms can experience chronic internal contamination even when external concentrations fluctuate across seasons, habitats and life stages.

### 3.4. Bioaccumulation, Trophic Transfer and Maternal Transfer

Two critical mechanisms responsible for the uptake of contaminants into aquatic organisms and reproductive risk include bioaccumulation and trophic transfer. Microplastics and nanoplastics may be ingested by organisms from lower trophic groups through the predator–prey relationship, which facilitates the transfer and transport of plastic particles and associated contaminants throughout the aquatic food web [44]. In addition, smaller sizes of particles are considered problematic due to ease of passing through biological barriers and the impact on embryos, larvae and juvenile organisms at specific developmental stages [42].

One of the contaminants for which trophic transfer is particularly relevant is per- and polyfluoroalkyl substances (PFAS), since the chemical stability of this pollutant facilitates long-term bioaccumulation and trophic transfer via contaminated water, sediments and prey [45]. The recent literature also suggests that maternal PFAS transfer occurs among aquatic mammalian, reptilian, fish, and bird species [46,47]. Evidence also indicates that microplastics and nanoplastics can be maternally transferred to offspring in some aquatic species. Due to their small size, particularly in the nano-scale range, these particles may cross biological barriers and accumulate in reproductive tissues, where they can subsequently be transferred to developing embryos [48]. Experimental studies in fish have reported the presence of plastic particles in eggs and offspring following parental exposure, suggesting a potential pathway for early-life-stage exposure and developmental toxicity [42]. Similarly, endocrine-disrupting chemicals such as phthalates and bisphenols can accumulate in aquatic organisms and food webs, increasing the risk of long-term reproductive effects when exposure overlaps with gonadal development, gametogenesis or early embryogenesis [3,14].

### 3.5. Factors Controlling Exposure Risk: Particle Properties, Species Traits and Environmental Conditions

The possibility of exposure in aquatic life forms is influenced by the physical and chemical characteristics of both the contaminant and the life form. For example, when it comes to microplastics and nanoplastics, the particle’s size, shape, polymer, electrical charge, the level of weathering and chemical additives will be important factors that determine their uptake, retention and interaction with the biological tissue and toxicities; small particles usually pose more risk due to the ability to penetrate the biological membrane [49,50]. Regarding the risk of PFAS exposure, chain length, functional groups, hydrophobicity, salinity, organic content and interaction with sediments are important factors, where long-chain PFAS tends to accumulate better than many short-chain PFAS [51]. In the case of EDCs, persistence, hydrophobicity, sedimentation and biological activity play significant roles [40].

Environmental alterations as a result of climate change could also affect contaminant exposure and toxicity in aquatic environments. Increased water temperature may alter the absorption, metabolism, and toxicity of contaminants as a result of increases in metabolic rate and physiological stress among aquatic species [52]. Changes in salinity, pH levels, dissolved oxygen, and hydrology due to climate change can further impact microplastic, PFAS, and endocrine-disruptor chemical transport, availability, and persistence. For example, increased temperature and UV light [53] will speed up plastic degradation into smaller particles.

Although there is general acceptance that species’ characteristics and environmental conditions affect exposure to contaminants, several questions remain. Some studies have shown increased uptake of contaminants in early life stages and greater sensitivity, but the degree of sensitivity differs among species, habitats, and contaminants. Species-specific traits strongly influence contaminant exposure and reproductive risk in aquatic animals. Feeding mode, habitat, trophic level, respiratory route, lipid content and reproductive stage determine whether uptake occurs mainly through water, sediment, prey or direct contact with contaminated particles. Early-life stages are especially sensitive because embryos and larvae have developing organs, high metabolic demand and limited detoxification capacity. Environmental factors such as temperature, salinity, pH, dissolved organic matter and hydrodynamics further modify contaminant transport, bioavailability and uptake.

## 4. Mechanistic Pathways Linking Emerging Contaminants to Reproductive Toxicity

The relationship between contamination and reproductive failure can be elucidated by identifying the cellular and molecular mechanisms by which the two variables relate to each other. Although individual contaminants may have identifiable molecular initiating events or dominant biological targets, adverse reproductive outcomes commonly arise through multiple downstream and interacting molecular, cellular, physiological, and behavioral responses. For microplastics, nanoplastics, PFAS, and endocrine-disrupting chemicals, the relative contribution of these responses depends on contaminant properties, exposure concentration and duration, developmental stage, and species-specific susceptibility. Microplastics, nanoplastics, PFAS, and endocrine-disrupting chemicals can impair reproductive function through several converging mechanisms of action. It has recently been confirmed by systematic reviews that there is microplastic toxicity variability according to shape, size, and polymer type with higher toxicity in cases where there is chronic exposure than acute exposure to the environmentally relevant concentration of microplastics [54]. In the same manner, PFAS mixtures have been observed to exhibit synergy on freshwater organisms [55].

An important consideration is that the same reproductive outcome can be achieved via various mechanisms based on the contaminant characteristics, exposure circumstances, and species properties. Fecundity reduction in female fish could occur due to oxidative stress-induced follicular atresia (which is typical for microplastics), due to hormonal imbalance caused by steroidogenesis disturbance from enzymes’ inhibition (which is typical for PFAS and phthalates), due to reduced production of vitellogenins due to estrogenic receptor antagonism (which is typical for some pesticides), or due to behavioral suppression of spawning behavior (which is typical for BPA and other neuroactive EDCs) [56,57,58]. Consequently, a study that reports only one of these mechanisms without excluding others provides an incomplete picture. Mechanistic research must therefore adopt a panel approach, measuring multiple pathway biomarkers simultaneously rather than focusing on a single hypothesized mechanism. Among these mechanisms, oxidative stress and endocrine disruption currently have the strongest experimental support across multiple contaminant classes and aquatic species. Numerous laboratory and field studies have consistently linked oxidative damage, altered antioxidant responses, hormonal imbalance, and disrupted steroidogenesis with impaired reproductive performance (Table 2) [59].

These mechanisms can be interrelated. Oxidative stress can impair mitochondrial function, while mitochondrial reactive oxygen species may further amplify intracellular oxidative damage. Mitochondrial dysfunction can promote apoptotic signaling and impair cellular energy homeostasis [60]. Under combined exposure, joint effects may be additive, synergistic, or antagonistic. Therefore, synergistic or antagonistic interactions should be concluded only after comparing observed mixture responses with predictions from an appropriate reference model, such as concentration addition or independent action [61].

**Figure 1:**
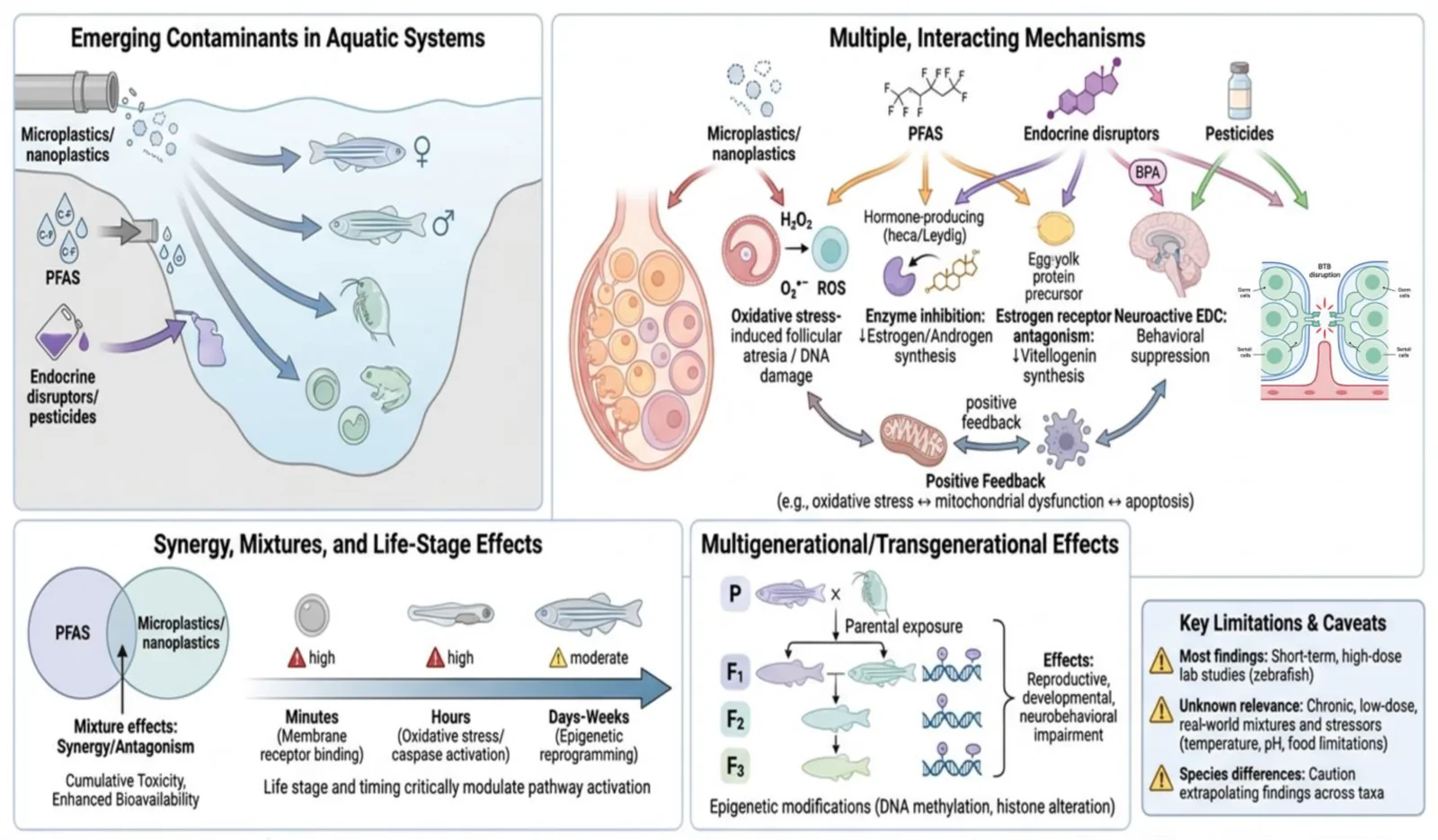
Mechanistic pathways linking emerging contaminants to reproductive toxicity in aquatic animals. Microplastics, nanoplastics, PFAS, endocrine disruptors, and pesticides impair aquatic reproduction through interacting mechanisms, including oxidative stress, mitochondrial dysfunction, endocrine disruption, enzyme inhibition, neuroendocrine disturbance, germ-cell barrier disruption, apoptosis, and epigenetic alteration. Mixture exposure and life-stage-specific sensitivity can amplify reproductive damage and contribute to reduced gamete quality, impaired development, and multigenerational effects. Abbreviations: PFAS, per- and polyfluoroalkyl substances; BPA, bisphenol A; ROS, reactive oxygen species; P, parental generation; F1–F3, filial generations; ↓, decrease. This figure is a conceptual illustration developed from the synthesis of findings reported in the reviewed literature and is intended to summarize the major mechanistic pathways and reproductive outcomes associated with emerging contaminant exposure in aquatic organisms.

In addition, mechanistic pathways are affected by timing and development stages. While certain reactions can take place within minutes, such as receptor binding, other reactions take hours, days, or weeks to develop, such as oxidative stress, activation of caspase, and epigenetic regulation. Early exposure during embryonic or larval stages is particularly risky due to the ongoing development of organs and detoxification mechanisms [62]. Adults that are undergoing gametogenesis actively are also susceptible since the dividing germ cells are quite susceptible to DNA damage and apoptosis. Hence, when assessing biomarkers, it is important to ensure that their measurement is consistent with the timing of exposure and the reproductive status of the organism [63]. Figure 1 presents a conceptual summary of the major mechanistic pathways linking emerging contaminant exposure to reproductive toxicity in aquatic animals, based on the synthesis of evidence from the reviewed literature.

Cross-generational outcomes should be interpreted according to the exposure status of each generation, because effects observed in descendants may result from direct exposure, parental effects, maternal transfer, or inherited biological alterations [64]. Multigenerational effects describe outcomes observed across two or more successive generations and may include experimental designs in which the parental generation and one or more descendant generations remain directly exposed [64]. Transgenerational effects should be reserved for effects persisting in descendants that have not experienced direct exposure to the initiating contaminant or contaminant carryover; therefore, the exposure status of F0, F1, and subsequent generations should be reported explicitly [64]. The two-generation zebrafish study of BPA by Chen et al. [65] illustrates the importance of distinguishing cross-generational reproductive outcomes from confirmed transgenerational inheritance. Similarly, Wang et al. [66] reported reproductive effects across generations in marine medaka following polystyrene microplastic exposure; however, such findings should be interpreted in relation to the documented exposure design and potential parental or maternal contributions. Epigenetic changes may contribute to cross-generational responses, but their detection alone does not establish transgenerational inheritance [64].

Currently, mechanistic insights are still based on experiments performed in short-term laboratory settings, using a small number of model organisms, especially zebrafish. Exposure to 50 and 500 µg/L of polystyrene microplastics in female zebrafish resulted in changes in the gonadosomatic index, reduction in fecundity, elevated ROS generation, hormonal imbalance, and disruption in reproductive physiology via SIRT1-dependent mechanisms [10]. At lower exposure levels, 2, 20, and 200 µg/L polystyrene microplastics delayed gonad maturation, decreased female fecundity, negatively regulated the HPG axis, and downregulated steroidogenesis-related genes in female fish [66]. Hence, mechanisms observed in laboratory conditions at higher exposures cannot be entirely applicable to lower environmental exposure regimes. Environmental stressors (e.g., warming, acidification) can also impact contaminant responses. In addition, mechanisms observed in zebrafish may not be entirely applicable to species using different reproductive strategies (e.g., salmonids, elasmobranchs, or crustaceans). Zebrafish findings should therefore be interpreted as useful mechanistic indicators rather than universally representative responses across aquatic taxa [67]. Although core pathways such as oxidative stress, endocrine disruption, mitochondrial dysfunction, apoptosis, and epigenetic alteration are broadly relevant, their reproductive consequences may differ among marine fish, elasmobranchs, crustaceans, mollusks, and amphibians because of taxon-specific differences in endocrine regulation, reproductive mode, developmental strategy, osmoregulation, and exposure routes [68,69,70]. Accordingly, field and mesocosm investigations across habitats, trophic levels, and species are needed to test whether laboratory-derived mechanisms occur under environmentally relevant exposure conditions [1,71,72].

**Table 2:** Mechanistic pathways linking emerging contaminants to reproductive toxicity in aquatic animals.

Mechanistic Pathway	Main Contaminants Involved	Key Biomarkers	Reported Results	Reproductive Consequence
Oxidative stress	Microplastics, nanoplastics, PFAS, pesticides, BPA, phthalates	ROS, MDA/lipid peroxidation, SOD, CAT and nitric oxide	Polystyrene microplastics increased ROS and apoptotic signals in female zebrafish and were associated with reduced fecundity, altered gonadosomatic index and hormonal imbalance [10]	Oxidative injury to gonadal cells, impaired oocyte development, reduced fecundity and potentially poorer offspring quality
Inflammation and immune stress	Microplastics, nanoplastics, PFAS, pesticides	Pro-inflammatory cytokines, NF-κB-related signaling and immune-response genes	Micro/nanoplastic exposure is linked with oxidative stress, inflammation, apoptosis and cellular injury in aquatic organisms [73,74].	Gonadal tissue injury, reduced reproductive condition and altered embryo–larval development
Mitochondrial dysfunction	Aged microplastics, nanoplastics, PFAS	Mitochondrial membrane potential, ROS, cytochrome-c and caspase-9/-3 signaling	Ding et al. [75] reported that aged polystyrene microplastics induced oxidative damage, reduced mitochondrial membrane potential, promoted cytochrome-c release and activated caspase-3/-9 signaling in early-life zebrafish	Reduced sperm motility, impaired oocyte competence, embryo toxicity and larval developmental defects
Endocrine disruption and HPG/HPGL-axis dysregulation	Microplastics, PFAS, BPA, phthalates, pesticides	GSI, estradiol, testosterone, cyp19b, esr2b, fshb, lhb, fshr, 17βhsd, cyp19a, lhr, vtg1 and vtg2	PFBS exposure reduced egg production and hatching rate, decreased gonadosomatic index by 73% in males and 50% in females, and disrupted hormone synthesis and HPGL-axis gene expression in zebrafish [76]	Altered gametogenesis, impaired spawning, reduced fecundity and abnormal sex-hormone balance
Apoptosis and ovarian-cell injury	Polystyrene microplastics	Expression of p53, bax, bcl2, cas3, and cas9, and TUNEL-positive ovarian cells	Gupta et al. [10] reported that polystyrene microplastic exposure altered SIRT1/p53-associated apoptotic gene expression and increased TUNEL-positive ovarian cells, together with disrupted oocyte maturation and impaired reproductive performance in female zebrafish	Ovarian-cell apoptosis, impaired oocyte maturation, reduced fecundity and fertilization, and decreased offspring hatching success
Oxidative DNA damage	Aged polystyrene microplastics	8-OHdG and ROS	Aged polystyrene microplastics increased 8-OHdG and ROS levels in early-life zebrafish, indicating oxidative DNA damage accompanied by mitochondrial dysfunction and apoptosis [75]	Impaired embryo–larval development, including reduced heart rate, body length and tail-coiling frequency
Epigenetic alteration	BPA	dnmt1 transcription, global gonadal DNA methylation, and gene-specific DNA methylation	BPA exposure reduced dnmt1 transcription, decreased global DNA methylation in the testes and ovaries, altered epigenetic- and reproduction-related gene expression, and reduced fertilization success in breeding zebrafish [77]	Epigenetic dysregulation in adult gonadal tissues and impaired fertilization.

Abbreviations: ROS, reactive oxygen species; SOD, superoxide dismutase; CAT, catalase; GPx, glutathione peroxidase; GST, glutathione-S-transferase; GSH, reduced glutathione; MDA, malondialdehyde; HPG axis, hypothalamic–pituitary–gonadal axis; HPGL axis, hypothalamic–pituitary–gonadal–liver axis; BPA, bisphenol A; PFAS, per- and polyfluoroalkyl substances; PFBS, perfluorobutane sulfonate/perfluorobutane sulfonic acid.

### 4.1. Oxidative Stress, Inflammation and Mitochondrial Dysfunction

Oxidative stress is one of the main cellular mechanisms by which emerging contaminants impair aquatic reproduction, because excessive reactive oxygen species (ROS) can damage lipids, proteins, DNA and mitochondrial membranes in gonadal and embryonic tissues. Microplastics and nanoplastics can increase ROS production, disturb antioxidant defense and trigger inflammatory responses, which may subsequently impair gametogenesis, oocyte maturation, sperm quality and embryo development [78]. In female zebrafish, polystyrene microplastic exposure increased oxidative and apoptotic responses that were associated with impaired reproductive performance [10].

Mitochondrial dysfunction provides another important link between contaminant exposure and reproductive toxicity because gametes and embryos require high mitochondrial activity for motility, fertilization, cleavage and early development. Aged polystyrene microplastics induced oxidative damage in early-life zebrafish, reduced mitochondrial membrane potential and promoted cytochrome-c release and caspase-3/-9 activation, indicating mitochondrial apoptosis as a key developmental toxicity pathway [75]. Similarly, PFAS exposure may contribute to reproductive impairment through oxidative stress and mitochondrial disruption; PFBS exposure in zebrafish induced oxidative stress, disrupted hormone synthesis and dysregulated hypothalamic–pituitary–gonadal–liver axis gene expression [76]. Therefore, oxidative stress, inflammation and mitochondrial dysfunction should be considered upstream mechanisms that connect contaminant exposure with endocrine disturbance, gonadal injury and reduced reproductive performance in aquatic animals.

### 4.2. Endocrine Disruption and Interference with the HPG Axis

Endocrine disruption is a central mechanism of reproductive toxicity because the hypothalamic–pituitary–gonadal (HPG) axis regulates sex steroid production, gametogenesis, spawning behavior and reproductive output in aquatic animals. Microplastics and nanoplastics can interfere with this axis by altering steroid hormone levels and the expression of genes involved in gonadotropin signaling, steroidogenesis and hormone receptors. In female zebrafish, polystyrene microplastic exposure reduced fecundity and altered reproductive endocrine markers, supporting a direct link between plastic-particle exposure and impaired HPG-axis regulation [10].

PFAS can also disrupt endocrine control of reproduction. Chronic exposure to perfluorononanoic acid (PFNA) in zebrafish caused dysfunction of the hypothalamic–pituitary–gonadal–liver (HPGL) axis, disturbed sex-hormone synthesis and produced adverse reproductive effects [79]. More recently, perfluorobutane sulfonate/perfluorobutane sulfonic acid (PFBS) exposure was shown to induce oxidative stress, disrupt hormone synthesis, dysregulate HPGL-axis gene expression and cause reproductive toxicity in both male and female zebrafish [76].

Classical endocrine-disrupting chemicals, including bisphenols, phthalates and pesticides, may act as hormone mimics, receptor antagonists or enzyme inhibitors, thereby disturbing estrogenic, androgenic and thyroid-related pathways in fish. Reviews on bisphenols in fish indicate that bisphenol A (BPA) and its analogs can disrupt hormone signaling and contribute to adverse reproductive and developmental outcomes [3]. Combined exposure is particularly relevant in aquatic systems; polyethylene microplastics and BPA significantly disrupted HPG-axis gene expression in zebrafish, with combined exposure producing stronger transcriptional effects than single-contaminant exposure [7]. Thus, contaminant-induced endocrine disruption provides a direct mechanistic bridge between environmental exposure and altered gametogenesis, spawning, fecundity and offspring development.

### 4.3. Genotoxicity, Apoptosis and Epigenetic Alterations

Genotoxicity and apoptosis provide important mechanistic links between contaminant exposure and reproductive impairment because DNA damage in gonadal cells, gametes or embryos can reduce fertilization success, embryo viability and offspring fitness. Microplastics and nanoplastics can induce DNA damage, chromosomal injury and altered gene expression, while oxidative stress and mitochondrial dysfunction may activate apoptotic pathways in reproductive tissues [80]. In zebrafish, micro- and nanoplastics have been associated with ovarian oxidative stress, DNA damage and increased apoptosis-related responses, suggesting that cellular injury may contribute directly to reduced reproductive performance [81].

Apoptotic signaling is particularly relevant because contaminant-induced germ-cell loss can impair gametogenesis and gonadal development. Hasan et al. [73] summarized that microplastics interfere with oxidative-stress and apoptotic pathways, leading to HPG-axis disruption, impaired steroidogenesis and gonadal dysfunction in aquatic organisms. Epigenetic disruption may also contribute to long-term and transgenerational reproductive effects, especially when exposure occurs during early development or active gamete formation. In breeding zebrafish, bisphenol A (BPA) exposure decreased dnmt1 transcription and reduced global DNA methylation in gonads, indicating that EDC-induced reproductive toxicity may involve altered epigenetic regulation as well as endocrine disruption [77]. Therefore, DNA damage, apoptosis and epigenetic modification should be considered key mechanisms through which emerging contaminants can reduce reproductive capacity across exposed individuals and subsequent generations.

### 4.4. Effects on Gametogenesis, Embryo Development and Larval Fitness

Emerging contaminants can impair aquatic reproduction by disrupting gametogenesis, fertilization, embryogenesis, and early larval performance. Microplastics may reduce gamete quality by altering gonadal structure, steroidogenesis, oxidative balance, and reproductive signaling pathways. In aquatic and other animal models, microplastic exposure has been associated with impaired gamete development, reproductive dysfunction, and adverse effects on embryos and offspring [82]. In zebrafish embryos, polyethylene microplastics were shown to penetrate the embryonic chorion and induce sublethal developmental cardiotoxicity, including reduced heart rate and altered expression of cardiac-development genes, indicating that early developmental stages are sensitive to plastic-particle exposure [83].

PFAS can similarly affect reproductive output and early developmental success by disrupting gonadal function, hormone synthesis, and oxidative balance. In adult zebrafish exposed to PFBS for 28 days, the highest tested concentration (14 μM) reduced egg production and hatching rate and decreased the gonadosomatic index in both sexes. PFBS accumulation was detected in testes and ovaries and was accompanied by impaired antioxidant defenses, altered sex-hormone profiles, dysregulation of hypothalamic–pituitary–gonadal–liver-axis genes, and histological damage to gonadal tissue [76]. Continuous low-level BPA exposure produced female-biased sex ratios and reduced sperm quantity and quality across exposed generations, while offspring from BPA-exposed parents showed delayed hatching, increased malformations, and higher mortality, with stronger developmental effects associated with paternal exposure [65].

Evidence from commercially important and non-model aquatic species confirms that reproductive and developmental toxicity is not limited to zebrafish. In the Pacific oyster (*Crassostrea gigas*), exposure to polystyrene microplastics impaired adult reproductive output, reduced gamete quality, lowered D-larval production, and affected offspring larval development [84]. Nanoplastics have also been shown to impair oyster gametes, embryos, and early free-living stages, while high-density polyethylene microplastics reduced Pacific oyster D-larval development and swimming activity in a particle-size-dependent manner [85,86]. In another commercially important bivalve, the scallop *Pecten maximus*, radiolabelled nanoplastics were taken up, distributed through tissues, and depurated under environmentally realistic exposure conditions, indicating potential exposure of edible shellfish tissues [87]. Non-model crustacean studies further support reproductive relevance: nanopolystyrene reduced *Daphnia magna* reproduction and increased neonate malformations, while micropolystyrene caused size-dependent toxicity in the marine copepod *Tigriopus japonicus* [88,89]. These findings indicate that commercially important shellfish, finfish, and non-model invertebrates should be included more explicitly when evaluating reproductive risk beyond zebrafish-based models.

### 4.5. Transgenerational and Population-Level Reproductive Consequences

Transgenerational effects are crucial since contamination during gametogenesis or embryogenesis can result in consequences for offspring that have not been directly exposed to the contaminants. In fish, micro- and nanoplastics can pass biological barriers, accumulate in gonadal tissue, and result in reproduction toxicity with concerns about the impact on the offspring through altered gamete quality and embryo development, and inherited changes in molecular levels [8]. Joint exposure might exacerbate such results; for instance, according to Luo et al. [90], joint exposure to polystyrene microplastics and arsenic resulted in reproduction toxicity and transgenerational effects in female zebrafish.

Persistent exposure to PFAS will result in lasting reproductive effects due to the persistent and bioaccumulative nature of the compounds, increasing the chance of exposure at sensitive life stages. PFAS-mediated reproductive toxicity results in gonadal toxicity, hormone disruption, and development abnormalities in model organisms, which include zebrafish and medaka [5]. Exposure of zebrafish during early life to environmentally relevant concentrations of perfluorooctane sulfonate (PFOS) caused reduced egg production by the adults. Both PFOS and perfluorobutane sulfonate/perfluorobutane sulfonic acid (PFBS) affected the development and behavior of the fish [13].

Reduced fecundity, poor quality gametes, sex differentiation issues, delayed hatching, reduced larval survival, and development abnormalities can lead to reduced recruitment rates and decreased population resilience at the population level. The endocrine-disrupting compounds, like bisphenols, phthalates, and pesticides, can impact fish HPG-axis function, reproductive behavior, gametes’ ability to reproduce, and population reproduction stability [91]. Nevertheless, there are several unanswered questions that need to be addressed. For example, long-term impacts of chronic low-dose exposures, contaminant mixtures, and the interactions with various environmental stressors, like warming and habitat destruction, are still not very well studied. Therefore, future studies have to focus on the multigenerational field-based approaches and environmentally realistic exposure scenarios.

## 5. Contaminant-Specific Reproductive Effects in Aquatic Animals

Contaminant-specific reproductive effects in aquatic animals are summarized in Table 3 to show how different emerging pollutants affect fertility, gamete quality, gonadal development and early offspring performance. Microplastics and nanoplastics are mainly linked with oxidative stress, endocrine disruption, gonadal injury, reduced fecundity and embryo–larval abnormalities, while PFAS are strongly associated with gonadal damage, sex-hormone disruption and adverse offspring development. Bisphenols, phthalates and pesticides further contribute to reproductive toxicity by interfering with steroidogenesis, vitellogenesis, gametogenesis and developmental programming, indicating that aquatic reproductive risk often results from overlapping endocrine, oxidative and developmental pathways.

### 5.1. Microplastics and Nanoplastics Interference

Microplastics and nanoplastics can impair aquatic reproduction by disrupting gonadal development, gametogenesis, steroid hormone balance, fertilization capacity and early offspring development. In fish, micro- and nanoplastics can cross biological barriers and accumulate in gonadal tissues, where they may induce oxidative stress, endocrine disturbance, apoptosis and transgenerational reproductive toxicity [8]. These effects are particularly important because reproductive endpoints such as fecundity, gonadosomatic index, gamete quality, hatching success and larval survival directly influence population renewal.

Experimental studies in zebrafish provide strong evidence for female reproductive impairment. Gupta et al. [10] exposed female zebrafish to polystyrene microplastics at 50 and 500 µg/L and observed altered gonadosomatic index, dose-dependent reduction in fecundity, increased reactive oxygen species, apoptotic signals, gonadal histological changes, disturbed estradiol/testosterone balance and altered HPG-axis-related gene expression. Similarly, Wang et al. [66] reported that exposure to polystyrene microplastics caused sex-specific reproductive disruption in marine medaka (Oryzias melastigma), including altered gonadal development and reproductive performance. In medaka, microplastic exposure has been associated with altered reproductive behavior, gonadal abnormalities, and impaired offspring development [94]. Studies in common carp and Nile tilapia have similarly reported oxidative stress, endocrine disruption, histopathological changes in gonadal tissues, and reduced reproductive performance following exposure to plastic particles [49].

Nanoplastics may pose additional risk because their smaller size increases their interaction with tissues and cells. Zhang et al. [95] demonstrated that polystyrene nanoplastics induced reproductive toxicity in female zebrafish by disrupting HPG-axis-related gene expression, altering sex-hormone levels, and reducing reproductive capacity, with additional endocrine and developmental effects observed in their unexposed offspring. Life-cycle exposure to differently charged polystyrene nanoplastics also affected zebrafish reproduction in a sex-specific manner and impaired F1 development, indicating that particle surface properties can influence reproductive outcomes and offspring fitness [96].

Microplastics and nanoplastics can also act as carriers or modifiers of other contaminants, thereby intensifying reproductive risk under mixed-exposure conditions. For example, co-exposure with polystyrene nanoplastics altered triclosan distribution in zebrafish tissues, exacerbated triclosan-induced spermatogenesis suppression in males, and increased embryonic mortality and larval malformations [93]. The enhanced toxicity observed during co-exposure is likely related to the ability of nanoplastics to adsorb and transport hydrophobic contaminants such as triclosan, thereby increasing their bioavailability and facilitating uptake into sensitive tissues. Owing to their small size and large surface-area-to-volume ratio, nanoplastics can act as contaminant carriers, promoting the accumulation and distribution of triclosan within reproductive organs and developing embryos [97]. Overall, current evidence indicates that microplastics and nanoplastics reduce reproductive performance through combined physical, endocrine, oxidative and developmental pathways, with particle size, charge, sex, life stage and co-contaminant exposure strongly influencing toxicity severity.

### 5.2. PFAS Interference

PFAS can impair aquatic reproduction because their persistence, bioaccumulation potential and endocrine activity allow exposure to overlap with gonadal development, gametogenesis, spawning and early embryogenesis. Current evidence indicates that PFAS-induced reproductive toxicity is mainly associated with gonadal injury, disruption of sex-hormone regulation and adverse effects on offspring development [5]. In fish, these effects are particularly important because changes in gonadosomatic index, egg production, hatching success and larval development directly affect reproductive output and population renewal.

Zebrafish studies provide strong evidence that both long-chain and short-chain PFAS can affect reproductive performance. Chronic exposure to perfluorononanoic acid (PFNA) caused higher PFNA accumulation in male gonads than female gonads, reduced male gonadosomatic index and female egg production, and decreased offspring hatching rate, suggesting that parental PFAS exposure can impair both adult reproduction and early developmental success [79]. Developmental exposure to environmentally relevant perfluorooctane sulfonate (PFOS) also reduced adult egg production in zebrafish, while both PFOS and perfluorobutane sulfonate/perfluorobutane sulfonic acid (PFBS) altered growth, organ development, anxiety-like behavior and liver lipid profiles [13]. The increasing use of short-chain PFAS has largely been driven by regulatory restrictions and phase-outs of several long-chain PFAS, including PFOS and perfluorooctanoic acid (PFOA), because of their persistence, bioaccumulation potential, and documented adverse health effects [98]. Short-chain PFAS are generally considered less bioaccumulative than their long-chain counterparts; however, they remain highly persistent in the environment and are increasingly detected in aquatic ecosystems worldwide [99].

Short-chain PFAS alternatives should not be assumed to be reproductively safe. PFBS exposure in adult zebrafish induced oxidative stress, disrupted sex-hormone regulation, dysregulated HPGL-axis-related gene expression, caused gonadal histopathological damage, and reduced reproductive performance [76]. PFBS also reduced antioxidant gene expression and triggered oxidative-stress-mediated apoptosis in zebrafish larvae, indicating that early-life exposure may compromise developmental fitness as well as later reproductive capacity [76]. Overall, PFAS exposure can reduce aquatic reproductive success through endocrine disruption, gonadal injury, oxidative damage and impaired offspring development.

### 5.3. Bisphenols, Phthalates and Pesticides Interference

Bisphenols, phthalates and pesticides are among the most studied endocrine-disrupting chemicals in aquatic reproductive toxicology because they can interfere with estrogenic, androgenic and steroidogenic pathways. Bisphenol A (BPA) and its analogs can alter vitellogenin production, sex steroid signaling, gonadal development and reproductive behavior in fish, indicating that bisphenols may affect both adult fertility and developmental competence [3]. Long-term exposure to environmentally relevant BPA concentrations induced gonadal, liver and kidney histopathological changes, altered growth and caused feminization-related effects in zebrafish, suggesting that developmental exposure to estrogenic pollutants can have persistent reproductive consequences [100].

Phthalates can also disrupt fish reproduction by altering endocrine activity and gamete production. In adult zebrafish, exposure to mono-(2-ethylhexyl) phthalate (MEHP), a major metabolite of di-(2-ethylhexyl) phthalate (DEHP), significantly decreased the number of ovulated eggs in females, reduced hepatic vitellogenin mRNA abundance, and increased estradiol, progesterone and cortisol levels, linking phthalate exposure with female reproductive dysfunction and stress-related endocrine disruption [6]. Previous evidence also indicates that DEHP exposure can impair fertilization ability through disturbed gonadal development, spermatogenesis, oogenesis and steroid biosynthesis in fish [6].

Pesticides represent another important reproductive risk because they can disrupt the hypothalamic–pituitary–gonadal–liver axis, inhibit steroid synthesis, alter aromatase activity, induce DNA damage and methylation changes, and promote oxidative stress and apoptosis. These interacting mechanisms can ultimately reduce fertility and offspring viability in fish [101]. In aquaculture-relevant species, insecticide exposure has also been linked with impaired endocrine regulation, disrupted gametogenesis and reproductive failure, showing that pesticide contamination is relevant not only to wild aquatic populations but also to farmed fish health [102]. Overall, bisphenols, phthalates and pesticides reduce aquatic reproductive performance mainly through endocrine disruption, altered steroidogenesis, gametogenic impairment and early developmental toxicity.

### 5.4. Mixture Toxicity and Combined Contaminant Exposure

In most cases, aquatic life is exposed to multiple pollutants at once instead of individual chemicals, which makes the study of combined toxicological effects particularly important in terms of reproductive health risks. Microplastics and nanoplastics can affect the toxicity of other pollutants by adsorbing chemicals from the surroundings onto their surface, altering the distribution and availability of the chemical, and thus increasing oxidative, hormonal, or developmental stress. Co-exposure studies have revealed that combined effects between particles and chemicals are either additive, antagonistic, or synergistic.

New insights from zebrafish studies support this concern. Polyethylene microplastics, together with BPA exposure, had a significant impact on gene expression of the hypothalamic–pituitary–gonadal axis, with a stronger response observed under conditions of combined exposure compared to individual exposure to contaminants [7]. In a similar way, the presence of polystyrene nanoplastics influenced the distribution of triclosan in zebrafish organs, causing more pronounced triclosan accumulation in the testes and liver of males, spermatogenesis inhibition, increased embryo death, and abnormalities in larvae [93]. These findings indicate that plastic particles can act not only as direct reproductive stressors but also as modifiers of other toxicants.

Mixture toxicity is also relevant for PFAS–microplastic interactions because both contaminant groups frequently co-occur in aquatic systems and share toxicity pathways such as oxidative stress, DNA damage and endocrine disruption (Table 4). Recent work in the freshwater sentinel species *Daphnia magna* has reported reproductive and developmental toxicity following co-exposure to PFAS and microplastics, underscoring the need to evaluate mixture effects rather than assess these contaminant groups separately [103]. Overall, combined contaminant exposure may better represent real aquatic conditions and should be incorporated into future reproductive studies using endpoints such as fecundity, gamete quality, gonadosomatic index, hatching success, larval survival and transgenerational effects.

### 5.5. Comparative Sensitivity Across Aquatic Taxa and Life Stages

Sensitivity to emerging contaminants can differ markedly among aquatic taxa because exposure routes, feeding behavior, habitat use, reproductive strategy, and developmental stage influence contaminant uptake, internal distribution, and biological response. Fish are among the most frequently studied aquatic groups; however, extrapolation from a single model species, particularly zebrafish, should account for differences in reproductive biology and life-history traits among taxa [67].

Zebrafish are small cyprinids with high fecundity, external fertilization, rapid development, and asynchronous ovarian development, making them useful for laboratory toxicity testing. Nevertheless, these characteristics are not representative of all fish species. Species with seasonal reproductive cycles and more synchronized gonadal development may have relatively narrow periods during which contaminant exposure can disrupt gametogenesis or spawning. Therefore, reproductive responses observed in zebrafish should not be directly generalized to fish with contrasting reproductive strategies without species-specific evidence [67].

Elasmobranchs generally have low reproductive output, internal fertilization, and relatively long reproductive cycles. These life-history traits may increase concern about contaminant-related reproductive impairment because population recovery can be slow when reproductive success declines. Although elasmobranchs are increasingly recognized as valuable indicators of marine pollution, mechanistic studies directly linking contaminant exposure with reproductive outcomes remain limited relative to those available for teleost fish [68].

Aquatic invertebrates should be considered separately because their exposure routes, physiology, and endocrine systems differ from those of fish. Bivalves are exposed to suspended particles through filter feeding, and Pacific oyster larvae can ingest both nano- and microplastic particles during sensitive developmental stages [70]. Crustaceans also require taxon-specific interpretation because their endocrine regulation involves ecdysteroids and methyl farnesoate rather than vertebrate sex-hormone pathways. Consequently, endocrine biomarkers and reproductive endpoints validated in fish cannot automatically be transferred to crustaceans [106]. Similarly, in Daphnia magna, chronic co-exposure to PFAS and polyethylene terephthalate microplastics impaired development, delayed maturation, and reduced growth, illustrating the sensitivity of aquatic invertebrates to contaminant mixtures [103].

Life stage at the time of exposure is an important determinant of reproductive hazard and should be assessed alongside species-specific sensitivity. Embryos, larvae, and juveniles may be particularly vulnerable because rapid organogenesis, physiological maturation, and sex differentiation occur during these periods, while protective detoxification and antioxidant systems are still developing [42]. During gonadal sex differentiation, endocrine-disrupting chemicals can interfere with gonadal development and sex differentiation in juvenile fish, potentially affecting sex ratios and later reproductive capacity [107]. Adults also remain vulnerable, particularly during gametogenesis; therefore, risk assessments based only on adult fish or a single model species cannot fully characterize reproductive susceptibility across life stages and aquatic taxa. While zebrafish remain valuable model organisms for mechanistic investigations, findings should be extrapolated cautiously to salmonids, elasmobranchs, and aquatic invertebrates because their reproductive biology, life-history traits, and exposure pathways differ substantially [67,68,106].

## 6. Biomarkers and Endpoints for Assessing Reproductive Toxicity

For the evaluation of reproductive toxicity in aquatic organisms, it is necessary to have both biomarkers as well as apical reproductive endpoints since molecular or hormone-related changes may sometimes fail to accurately assess the impacts on the fertility and survival rate of the offspring. The usual methods used for testing fish reproduction include use of endocrine biomarkers like vitellogenin, sex hormones, secondary sexual characters, gonadosomatic index, and histopathological examination of the gonads, coupled with apical endpoints of reproductive tests that include fecundity, sperm fertilization capacity, successful embryo hatching, and viability of F1 generation.

### 6.1. Hormonal and Endocrine Biomarkers

Hormone and endocrine markers play a significant role in determining reproductive disruptions due to contaminants in aquatic animals. Some of the hormone markers used in fish are vitellogenin, estradiol, testosterone, 11-ketotestosterone, gonadosomatic index, and the presence of secondary sex characteristics and genes related to the hypothalamic–pituitary axis [108].

Vitellogenin is particularly useful because it is an estrogen-inducible yolk precursor protein, and abnormal induction in males or juveniles is widely used as evidence of estrogenic endocrine disruption [109]. However, endocrine biomarkers should be interpreted together with reproductive endpoints such as fecundity, gonadal histopathology and fertilization or hatching success, because hormonal changes alone may not fully predict reproductive impairment [110].

### 6.2. Oxidative Stress, Inflammation and Mitochondrial Biomarkers

Oxidative stress and inflammatory and mitochondrial biomarkers provide early evidence of contaminant-induced cellular injury before clear reproductive failure becomes visible. Common biomarkers include reactive oxygen species (ROS), superoxide dismutase, catalase, glutathione peroxidase, glutathione-S-transferase, reduced glutathione and malondialdehyde, which reflect antioxidant defense, redox imbalance and lipid peroxidation status [111]. Gupta et al. [10] showed that polystyrene microplastic exposure in female zebrafish increased ROS generation and apoptotic responses, alongside reduced fecundity, altered gonadosomatic index and disrupted reproductive hormone balance.

Mitochondrial biomarkers are especially relevant because gametes, embryos and larvae depend on mitochondrial function for energy production, fertilization and early development. Ding et al. [75] reported that aged polystyrene microplastics induced oxidative damage in early-life zebrafish, reduced mitochondrial membrane potential and activated cytochrome-c/caspase-9/caspase-3 signaling, indicating mitochondrial apoptosis as an important toxicity pathway. Similarly, Santhi et al. [76] found that perfluorobutane sulfonate/perfluorobutane sulfonic acid (PFBS) exposure induced oxidative stress, disrupted hormone synthesis, dysregulated hypothalamic–pituitary–gonadal–liver axis gene expression and caused reproductive toxicity in both male and female zebrafish. Therefore, oxidative stress, inflammation and mitochondrial dysfunction should be interpreted together with reproductive endpoints such as gonadal histology, gamete quality, fecundity, hatching rate and larval survival.

### 6.3. Gonadal Histology and Gamete-Quality Endpoints

Gonadal histology provides direct evidence of structural reproductive damage and should be interpreted alongside functional gamete-quality endpoints. In fish, histological assessment commonly includes ovarian follicle stage distribution, oocyte maturation, oocyte atresia, follicular-layer disruption, testicular organization, spermatogenic stage progression and degeneration of germinal tissues; these endpoints are also recognized in fish reproduction assays together with fecundity, fertility, gonadosomatic index and endocrine biomarkers [112].

Gamete-quality endpoints strengthen histological interpretation because structural gonadal damage does not always translate directly into reproductive failure. For males, useful endpoints include sperm concentration, motility, viability, morphology, DNA integrity and fertilizing ability, while female endpoints include oocyte maturation, oocyte-stage composition, germinal vesicle breakdown, egg production, fertilization success and egg quality. Gupta et al. [10] showed that polystyrene microplastic exposure in female zebrafish altered ovarian morphology and oocyte-stage distribution, together with reduced fecundity, altered gonadosomatic index, increased ROS and disrupted hormonal balance.

PFAS studies also support the value of combining gonadal and gamete-related endpoints. Santhi et al. [76] reported that perfluorobutane sulfonate/perfluorobutane sulfonic acid (PFBS) exposure reduced egg production and hatching rate in zebrafish and decreased the gonadosomatic index by 73% in males and 50% in females, while also assessing gonadal histological changes, antioxidant status and sex steroid hormones. Therefore, gonadal histology and gamete-quality endpoints should be used together to connect contaminant-induced tissue injury with biologically meaningful reproductive outcomes.

### 6.4. Embryo, Larval and Offspring-Fitness Endpoints

Embryo, larval and offspring-fitness endpoints are essential because reproductive toxicity may appear after fertilization, even when adult biomarkers show only moderate changes. Key endpoints include fertilization rate, embryo survival, hatching rate, hatch timing, malformation frequency, heart rate, body length, yolk-sac absorption, swimming behavior, larval survival and F1 reproductive performance.

As previously discussed, PFAS-related studies also show the value of offspring endpoints. PFBS exposure significantly reduced egg production and hatching rate in zebrafish, while decreasing gonadosomatic index in females [76]. Developmental exposure to environmentally relevant perfluorooctane sulfonate (PFOS) reduced adult egg production, whereas both PFOS and PFBS altered growth, organ development, anxiety-like behavior and liver lipid profiles, showing that early-life exposure can influence later-life fitness [13]. BPA exposure has also been linked with reduced larval development and survival, particularly through parental exposure effects, supporting the need to include multigenerational offspring endpoints in aquatic reproductive toxicology [65].

## 7. Ecological and Aquaculture Implications

### 7.1. Consequences for Wild Fish Populations

At the population level, reproductive toxicity from emerging contaminants can reduce recruitment by lowering fecundity, fertilization success, embryo viability, hatching rate and larval survival. These effects are especially important in wild fish because even modest reductions in egg production or offspring survival may weaken population renewal when exposure is chronic or combined with other stressors such as warming, hypoxia and habitat degradation. Brander et al. (2026) emphasized that synthetic chemicals, including microplastics and PFAS, can interact with climate-related stressors to reduce fertility and fecundity and may contribute to multigenerational reproductive harm across wildlife taxa [1].

Wild fish populations may also be affected through endocrine disruption, altered sex differentiation and impaired reproductive behavior. Reviews on EDCs in aquatic systems indicate that these compounds can disturb reproductive physiology in fish, although field-level links between contaminant exposure and population decline are often harder to establish than laboratory effects because wild populations are exposed to multiple stressors simultaneously [14,71]. Similarly, fish inhabiting contaminated estuaries and industrialized waterways have exhibited reproductive abnormalities, impaired gonadal development, and reduced recruitment success linked to elevated contaminant burdens [113]. Microplastics and nanoplastics add further concern because they can impair early-life stages, disrupt endocrine function and reduce fertility-related outcomes, while PFAS and other persistent contaminants may produce developmental and multigenerational effects that influence long-term population resilience [114]. Therefore, wild fish risk assessment should integrate reproductive endpoints across life stages, including adult fecundity, gamete quality, embryo development, larval survival and F1 reproductive performance, rather than relying only on short-term mortality or adult tissue biomarkers.

### 7.2. Implications for Shellfish, Crustaceans and Aquatic Invertebrates

The shellfish, crustaceans, and other invertebrates that inhabit the aquatic environment are highly susceptible to new contaminants due to the filtration capabilities of many species, feeding on particulates, being sediment or bottom dwellers and thus accumulating pollutants from these environments. The importance of bivalves is related to the capacity of these animals to bioaccumulate microplastics, PFAS, and endocrine-disrupting chemicals from the water and particulates, which makes contaminant exposure relevant to reproduction, recruitment of larvae and population viability. Studies on monitoring of PFAS revealed the ability of bivalves to accumulate significant amounts of PFAS in their tissue; for example, PFOS was found in concentrations of 125.9 ng/g wet weight in Mediterranean mussels (Mytilus galloprovincialis) of north-central Portugal estuaries [115].

There is emerging evidence to suggest that these pollutants are capable of interfering with invertebrate reproduction via gonad disruption, oxidative stress, endocrine effects, and developmental impairment of their offspring. Smolarz et al. [116] found that environmentally relevant perfluorotetradecanoic acid exposure produced histopathological effects in the mussel *Mytilus trossulus*, such as gill edema, atrophy of digestive glands, and stimulation of gonads, with possible implications for respiration, digestion, and reproduction. In crustaceans, endocrine-disrupting chemicals may interfere with hormonal control of gonadal growth, sexual differentiation, offspring development, molting and metabolism, making reproductive assessment more complex than in fish because reproduction and molting are physiologically linked [69]. Therefore, shellfish and crustacean studies should include endpoints such as gametogenic stage, gonadal histology, spawning success, larval development, settlement, survival and offspring fitness, rather than relying only on adult accumulation or biochemical biomarkers.

### 7.3. Relevance to Aquaculture Productivity and Reproductive Management

In aquaculture, reproductive toxicity from microplastics, nanoplastics, PFAS and EDCs can reduce productivity by impairing broodstock quality, gamete performance, fertilization success, embryo development, hatching rate and larval survival. This is important because hatchery success depends on predictable spawning, high-quality eggs and sperm, and strong larval performance; therefore, contaminant-induced reproductive disruption may reduce seed production and increase production losses. Microplastics are relevant to aquaculture because they occur in fisheries and aquaculture systems and may affect physiological processes linked with productivity, while EDCs and pharmaceuticals can interfere with reproductive biology in aquatic fauna [14].

Emerging contaminants may also complicate reproductive management through chronic exposure from water, feed, sediments, biofilms and plastic-based culture materials. Microplastics and nanoplastics may impair reproduction through oxidative stress, endocrine disruption, gonadal injury and altered embryo–larval development, whereas PFAS contamination in aquaculture feeds and fish tissues raises concern for both fish health and food-safety monitoring [73,117]. Therefore, aquaculture monitoring should include reproductive endpoints such as gonadosomatic index, sex steroids, sperm motility, egg quality, fertilization rate, hatching success, larval deformities and early survival, together with contaminant screening in water, feed and broodstock tissues.

### 7.4. Food-Web Transfer and Ecosystem-Level Reproductive Risk

Food-web transfer is important because emerging contaminants can move from plankton, benthic organisms and small prey species to higher trophic fish, predators and humans. Microplastics can be ingested by lower trophic organisms and transferred through prey–predator interactions, while their surfaces may also carry plastic additives, PFAS, pesticides and metals, increasing the potential for combined toxic effects across food webs [72,105]. PFAS are of particular concern because recent global evidence shows trophic magnification in aquatic and terrestrial food webs, with an average trophic magnification factor of about 2, meaning concentrations can increase at higher trophic levels [118].

Ecosystem-level reproductive risk arises when contaminant transfer reduces the reproductive capacity of multiple connected species rather than a single exposed population. Microplastic exposure has been linked with reduced feeding, growth, survival and reproductive capacity in aquatic species, which may alter predator–prey interactions and weaken population recruitment [119]. PFAS transfer through aquatic food webs may also expose predatory fish and wildlife during sensitive reproductive stages, increasing the risk of endocrine disruption, embryo toxicity and offspring effects. A recent synthesis on PFAS in fish emphasized that PFAS occurrence, internal accumulation and trophic transfer are key processes for understanding exposure and ecological risk in aquatic systems [120]. Therefore, ecosystem-level assessment should integrate contaminant accumulation, trophic transfer, reproductive biomarkers and population endpoints to better predict long-term effects on aquatic biodiversity and food-web stability (Table 5).

## 8. Risk Assessment and Regulatory Challenges

### 8.1. Limitations of Single-Contaminant Toxicity Testing

Single-contaminant toxicity testing has limited ecological relevance because aquatic animals are usually exposed to mixtures of microplastics, nanoplastics, PFAS, endocrine-disrupting chemicals, pesticides, metals and pharmaceuticals rather than one pollutant alone. Standardized assays such as the OECD Fish Short-Term Reproduction Assay are valuable because they assess fecundity, vitellogenin, secondary sex characteristics and gonadal histopathology in mature fish, but they mainly evaluate individual chemicals over a short exposure period.

This limitation is important because mixed contaminants can produce additive, synergistic or antagonistic effects that are not predictable from single-chemical data. Combined toxicity testing better reflects environmental exposure, where chemical mixtures co-exist and may affect multiple biochemical, endocrine and developmental pathways simultaneously. PFAS–microplastic co-exposure can also modify contaminant transport, bioavailability and biological responses in aquatic organisms [124]. Therefore, reproductive toxicity studies should move beyond single-compound designs and include environmentally realistic mixtures, chronic low-dose exposure, early-life sensitivity and multigenerational endpoints such as fecundity, gamete quality, hatching success, larval survival and offspring fitness.

### 8.2. Experimental Concentrations Versus Environmentally Observed Levels

Experimental exposure concentrations should be interpreted in relation to measured environmental concentrations because high-dose laboratory studies may be useful for hazard identification but may not directly represent field exposure [125,126]. For microplastics and nanoplastics, comparison is complicated because environmental studies often report particle number, whereas laboratory studies may report mass concentration, particle size, polymer type, shape, and pristine or aged particles; available exposure–hazard comparisons indicate that many laboratory concentrations exceed typical open-water levels [125,126]. PFAS have been detected in aquatic environments, wastewater, and drinking-water sources at low ng/L levels, while substantially higher concentrations have been reported near contaminated point sources such as industrial areas, wastewater treatment plants, and firefighting-foam-impacted sites [127,128,129]. For endocrine-disrupting chemicals such as BPA, phthalates, and pesticides, low-dose experiments may be environmentally relevant when supported by monitoring data, whereas high-dose experiments should be interpreted mainly as mechanistic or hazard-identification evidence [130,131,132]. Therefore, future reproductive-toxicity studies should justify selected concentrations using measured environmental data and include chronic low-dose, mixture, and life-stage-specific exposure designs [132].

### 8.3. Low-Dose, Chronic and Non-Monotonic Endocrine Responses

Low-dose and chronic exposure are critical issues in aquatic reproductive toxicology because endocrine-active contaminants can affect hormone signaling at concentrations that do not cause obvious mortality. Traditional high-dose toxicity testing may not reliably predict low-dose endocrine effects, particularly when chemicals interfere with receptor binding, steroidogenesis or developmental programming [133].

Non-monotonic dose responses further complicate risk assessment because biological effects may be stronger at low or intermediate concentrations than at higher concentrations. This is especially relevant for EDCs such as bisphenol A (BPA), where low-dose exposure has been associated with altered endocrine signaling and reproductive effects in fish and other vertebrates [133,134].

In zebrafish, environmentally relevant BPA exposure disrupted reproductive processes and reduced dnmt1 expression and global DNA methylation in gonads, showing that low-dose exposure can affect both endocrine and epigenetic regulation [77]. Therefore, reproductive risk assessment should include chronic low-dose exposure designs, sensitive endocrine biomarkers and life-stage-specific endpoints rather than relying only on high-dose acute toxicity tests.

### 8.4. Mixture Toxicity and Environmentally Realistic Exposure Models

Mixture toxicity poses another important threat due to interactions between pollutants in the aquatic environment that might be additive, synergistic or antagonistic, hence making it hard to extrapolate reproductive risks based on the effects of individual pollutants. Both microplastics and nanoplastics are expected to modify the toxicity of pollutant mixtures through adsorption of PFAS, pesticides, metals and endocrine disruptors, thus affecting the movement, bioavailability and tissue accumulation of these contaminants. PFAS and microplastic co-exposure have been shown to increase the toxicity of these pollutants since co-exposure in Daphnia magna caused developmental failure, delayed sexual maturation and inhibited somatic growth, where the combination was found to be 59% additive and 41% synergistic without any antagonistic interactions [103]. Therefore, future aquatic reproductive studies should use environmentally realistic mixtures, chronic low-dose designs and endpoints such as fecundity, gamete quality, hatching success, larval survival and F1 reproductive performance.

### 8.5. Standardization of Microplastic, Nanoplastic and PFAS Detection Methods

Uniform testing protocols are important because sampling, extractions, particle size cut-off, polymer analysis, and contamination prevention vary widely between micro- and nanoplastics research, leading to incompatibility in data comparison. Risk assessments on plastic particles should include information on particle size and shape, polymer type, degree of aging, surface chemistry and concentration, as these factors significantly affect absorption and toxicity; contemporary studies highlight the importance of developing decision-ready analysis tools [135,136]. PFAS monitoring also demands standardization of methodologies for various types of water, sediment, wastewater, biota, and biosolids since the concentrations and risk assessment depend upon the matrix type and class of compounds. The U.S. EPA states that the PFAS analytical methods for drinking water, groundwater, surface water, wastewater, solid, sediments, biota and biosolids are currently being developed, whereas recent PFAS monitoring guidance highlights the expansion of PFAS analysis from PFOS to many other PFAS compounds [137]. Consequently, future research on reproductive risk assessment would do well to incorporate chemical measurements along with biological indicators such as hormonal measures, gonadal histopathology, fecundity, hatching, and larval survival.

### 8.6. Translating Laboratory Findings to Field-Level Reproductive Risk

**Figure 2:**
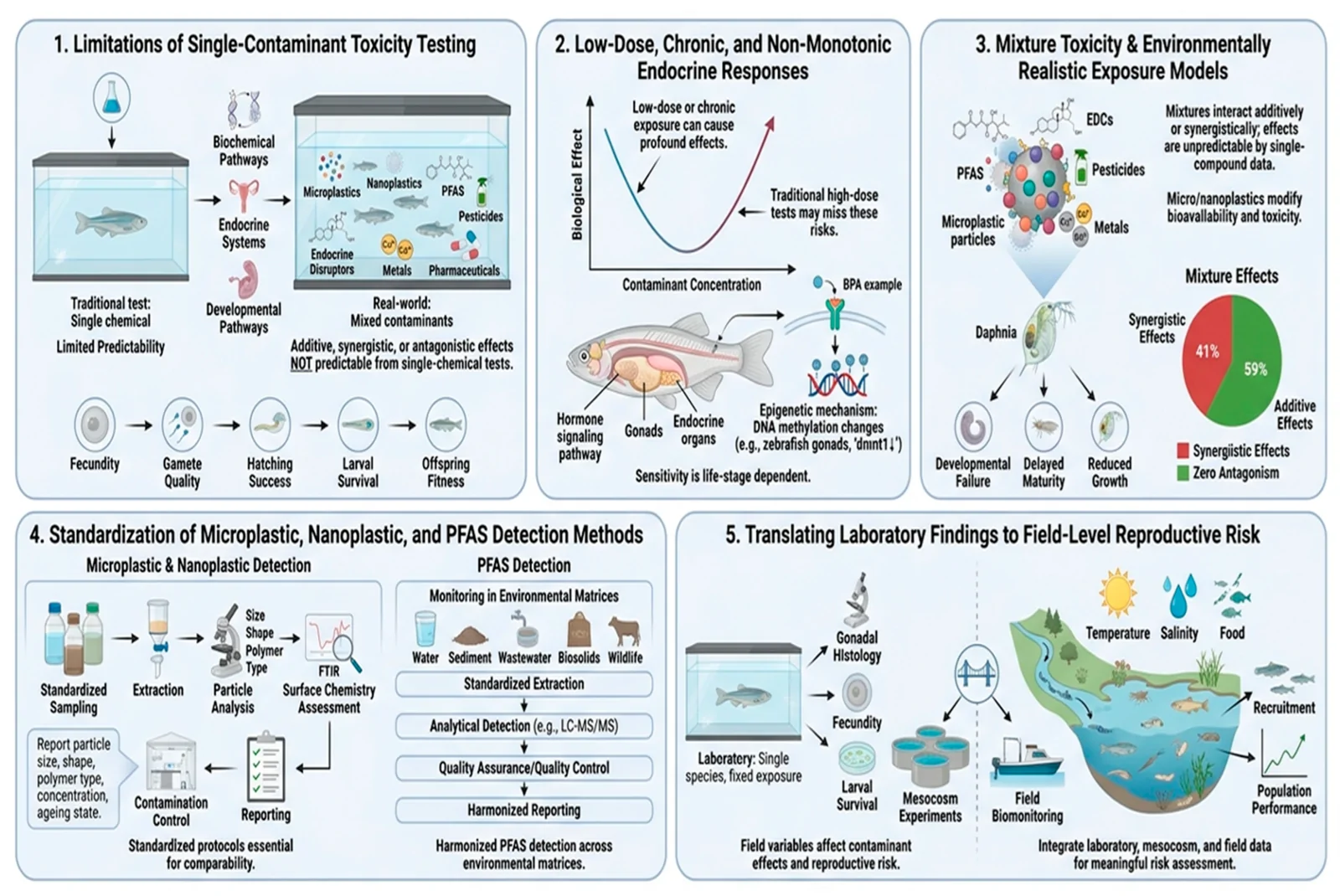
Risk assessment and regulatory challenges in aquatic reproductive toxicology. Key limitations include reliance on single-contaminant tests, uncertainty in predicting mixture toxicity, low-dose and non-monotonic endocrine responses, inconsistent contaminant-detection methods, and limited translation of laboratory findings to field-level reproductive risk. Improved ecological relevance requires standardized contaminant detection, mixture-based assays, chronic low-dose exposure models, mesocosm studies, and field monitoring. Abbreviations: PFAS, per- and polyfluoroalkyl substances; EDCs, endocrine-disrupting chemicals; BPA, bisphenol A; LC–MS/MS, liquid chromatography–tandem mass spectrometry.

Relating laboratory toxicity tests to reproductive risks in the field is problematic because laboratory tests usually employ controlled exposure environments, single species and constant pollutant levels, while natural environments feature varying concentrations of contaminants, changing temperatures, salinities, food availability, and species interactions. Microplastic effects in aquatic ecosystems are strongly context-dependent, with outcomes influenced by environmental setting, particle properties and organism life stage, making direct extrapolation from laboratory results uncertain [72]. The major challenges associated with translating laboratory findings into field-level reproductive risk, together with the corresponding research priorities, are summarized in Figure 2.

For PFAS and other persistent contaminants, field risk assessment is further complicated by bioaccumulation, trophic transfer and long-term exposure across sensitive reproductive stages. EPA notes that PFAS occur in water, air, fish and soil and persist in the environment, supporting the need to connect laboratory endpoints with field monitoring of exposure and biological effects. Therefore, future studies should combine laboratory assays, mesocosm experiments, field biomonitoring and population-relevant endpoints such as fecundity, hatching success, larval survival, recruitment and multigenerational reproductive performance. Hence, laboratory assays should be integrated with mesocosm experiments, field biomonitoring and population-relevant endpoints, including fecundity, hatching success, larval survival, recruitment and multigenerational reproductive performance.

## 9. Mitigation Strategies and Future Research Priorities

### 9.1. Reducing Contaminant Inputs into Aquatic Environments

Source control is the best protection for aquatic reproductive health. Once microplastics, PFAS, and EDCs enter water bodies, sediments, and food webs, their removal becomes technically challenging and costly. For plastic-derived contaminants, mitigation should focus on reducing unnecessary plastic use, improving circular product design, increasing recycling, preventing industrial pellet loss, controlling textile fibers and tire-wear particles, and reducing leakage from fishing and aquaculture plastics. OECD identifies four key policy pillars for plastic control: reducing production and demand, designing for circularity, improving recycling and closing leakage pathways [9].

For PFAS and EDCs, source reduction should prioritize industrial discharge control, wastewater permitting, landfill-leachate management, safer chemical substitution and phase-out of high-risk uses such as PFAS-containing firefighting foams. EPA’s PFAS roadmap emphasizes limiting PFAS releases through monitoring, waste management, destruction/disposal guidance and regulatory controls on contaminated materials [138]. Therefore, prevention-based policies and cleaner production are essential to reduce chronic contaminant exposure and protect reproduction in aquatic animals.

### 9.2. Improved Wastewater, Runoff and Aquaculture-System Management

Improved wastewater and runoff management is essential because treatment plants, stormwater systems and aquaculture facilities can act as pathways for microplastics, PFAS and EDCs into aquatic environments. Advanced treatment options such as membrane filtration, adsorption, coagulation, oxidation and bio-based treatment can reduce contaminant release, but their effectiveness depends on the contaminant type, particle size, water chemistry and operational conditions. The major treatment technologies, management strategies, advantages, limitations, and target contaminants are summarized in Table 6. PFAS removal can involve adsorption, membrane filtration, electrochemical oxidation and biological degradation, although short-chain PFAS removal, material regeneration and large-scale application remain major challenges [139,140].

Stormwater and agricultural runoff should be controlled through sediment traps, constructed wetlands, vegetated buffer zones, runoff retention systems and improved management of plastic waste and pesticide use. In aquaculture, mitigation should include reducing degradation of plastic-based equipment, improving feed and water quality, monitoring sediment contamination, and preventing contaminated effluent release from ponds, cages and hatcheries. Microplastic removal can be improved through coagulation and filtration-based approaches, with removal efficiency strongly influenced by particle type, concentration and wastewater characteristics [140].

### 9.3. Monitoring Programs Using Reproductive Biomarkers

Monitoring programs should combine contaminant measurements with reproductive biomarkers to detect early reproductive stress before population-level effects become visible. Useful endpoints include sex steroids, vitellogenin, gonadosomatic index, gonadal histopathology, HPG/HPGL-axis gene expression, oxidative-stress markers, sperm motility, egg quality, fertilization rate, hatching success and larval survival. These biomarkers have relevance due to the fact that microplastics affect reproduction via endocrine disruption, oxidative stress, and gametogenesis whereas PFAS affect reproductive performance via gonadotoxicity, sex-hormone disruption, and effects on offspring development [73].

Biomonitoring for reproduction should involve linking mechanistic biomarkers with functional parameters such as fecundity, gamete quality, embryo survivability, larval survivability, and offspring fitness. This integrated approach matters because pharmaceuticals and EDCs can disrupt reproductive biology in aquatic animals. Molecular responses alone might not be enough to predict reproductive issues from chronic or mixed-contaminant exposures [14].

### 9.4. Long-Term, Multigenerational and Life-Cycle Studies

Multigenerational studies are important since short-term tests could not identify possible reproductive problems occurring at certain development stages. Multigenerational effects occur from microplastics in fish, but evidence for true transgenerational effects is yet to be found. Exposure to PFAS has been associated with gonad injury, hormone alterations, and problems in the development of offspring [5]. The future studies must involve parental, F1, and F2 generations by observing such aspects as fecundity, gamete quality, fertilization efficiency, embryo survival, hatchability, larvae survival, sex ratio, growth, and reproduction ability.

### 9.5. Integration of Omics, Field Data and Reproductive Performance Endpoints

**Figure 3:**
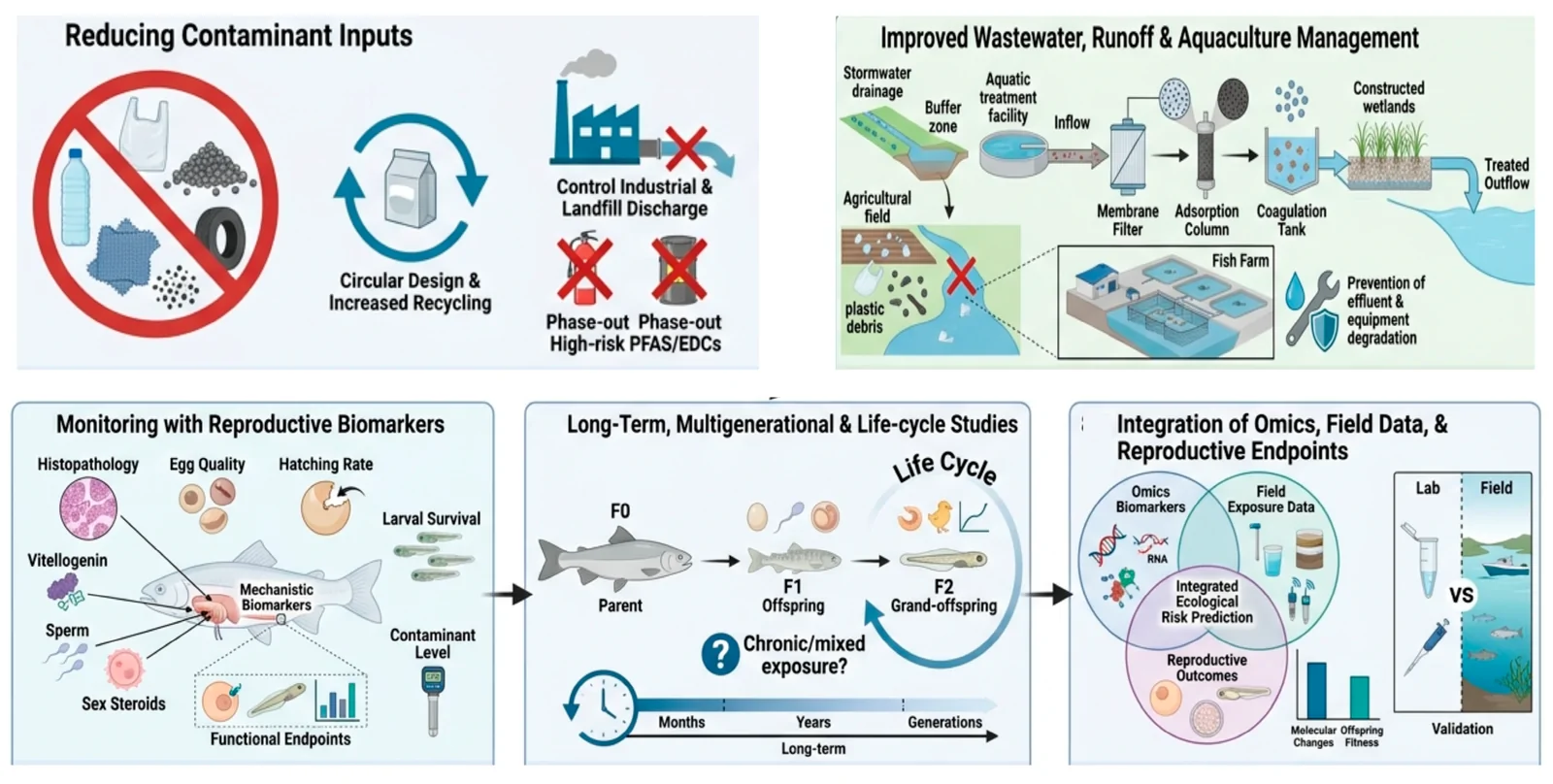
Mitigation strategies and future research priorities for protecting aquatic reproductive health. The figure shows important steps to lower reproductive risks from microplastics, PFAS, and EDCs. These include controlling sources, using circular design, improving water and runoff treatment, managing aquaculture, and monitoring reproductive biomarkers.

Further research should incorporate omics-based biomarkers together with information regarding exposure and reproductive performance in order to link early molecular perturbations to ecologically relevant endpoints. Transcriptomics, metabolomics, proteomics and epigenomics could detect perturbed pathways like steroidogenesis, oxidative stress, lipid metabolism, mitochondrial function, apoptosis and endocrine signaling; however, these perturbations have to be supported by fecundity, gamete quality, fertilization success, hatchability, larval viability and offspring fitness. Multi-omics approaches are increasingly useful in aquatic toxicology because they help explain contaminant effects across molecular, cellular and organismal levels [146]. The major future research priorities and integrated mitigation strategies for protecting aquatic reproductive health are summarized in Figure 3.

Field validation is essential because laboratory exposure does not fully represent fluctuating mixtures, environmental chemistry, species interactions and life-stage sensitivity in natural ecosystems. For microplastics, PFAS and EDCs, omics data should therefore be combined with contaminant monitoring in water, sediment, feed and tissues, together with reproductive endpoints that directly reflect population risk. This integrated design can improve risk prediction and support biomarker-based monitoring for aquatic reproductive health [5,73]. Future studies should also increase taxonomic coverage beyond model species and integrate multi-omics approaches with reproductive and population-level endpoints to improve ecological risk assessment.

## 10. Conclusions

Microplastics, nanoplastics, PFAS, and EDCs constitute novel threats to the reproductive health of aquatic life. Scientific studies have shown that these compounds may affect reproduction via related mechanisms such as oxidative stress, endocrine disruption, mitochondrial dysfunction, gonad damage, disrupted gamete formation, and developmental abnormalities. Across fish and other aquatic organisms, exposure has been associated with reduced reproductive performance, impaired offspring development, and possible consequences for population sustainability. PFAS are commonly associated with altered hormone regulation, gonadal dysfunction, and developmental effects, whereas microplastics and nanoplastics have been linked with oxidative, endocrine, reproductive, and offspring-related effects across aquatic taxa. However, the available evidence is still dominated by controlled laboratory studies, particularly short-term experiments using zebrafish and individual contaminants. Therefore, the magnitude, persistence, and ecological relevance of reported reproductive effects across diverse aquatic taxa and environmentally realistic exposure conditions remain uncertain. Reproductive risk assessment should move beyond short-term, single-contaminant testing by incorporating environmentally realistic mixtures, chronic low-dose exposures, early-life-stage sensitivity, and multigenerational endpoints. Standardized chemical characterization, integrated reproductive biomarkers, omics-based approaches, and validation through mesocosm and field studies are needed to improve prediction of population-level reproductive risks and guide protective strategies for aquatic ecosystems.

## Figures and Tables

**Table 1:** Major sources and environmental pathways of endocrine-disrupting chemicals (EDCs) in aquatic ecosystems.

Pathway	Major EDCs	Relative Importance	Main Concern
Municipal wastewater [32]	Bisphenols, pharmaceuticals, synthetic hormones, phthalates	High	Continuous discharge and incomplete removal during treatment
Agricultural runoff [33]	Pesticides, veterinary pharmaceuticals, hormones	High	Seasonal pulses and widespread contamination of surface waters
Industrial effluents [34]	Bisphenols, phthalates, specialty chemicals	Moderate–High	Localized high concentrations near discharge points
Landfill leachate [35]	Bisphenols, PFAS, plastic additives	Moderate	Long-term contaminant release to groundwater and surface waters
Plastic-associated chemical release [36]	Bisphenols, phthalates, additives	Moderate	Chronic diffuse contamination and co-exposure with microplastics

**Table 3:** Reported reproductive and developmental effects of microplastics, nanoplastics, PFAS and EDCs in aquatic animals.

Contaminant/Stressor	Chemical Characteristics	Species/Model, Stage and Sex	Exact Concentration/Dose	Concentration/Dose	Main Reproductive/Developmental Results	Proposed Mechanism/Biomarkers	Ref.
Polystyrene microplastics	Carboxylate-modified commercial PS-MPs; nominal mean size, 0.5 µm; hydrodynamic diameter, 529.3 ± 161.9 nm; zeta potential, −24.9 ± 0.61 mV; pristine particles	Sexually mature adult female zebrafish, *Danio rerio*	50 and 500 µg/L PS-MPs	60 days	PS-MPs accumulated in ovarian tissue and significantly increased GSI at 500 µg/L. Exposure reduced egg production and spawning, lowered fertilization at 500 µg/L, reduced F1 hatching success, altered ovarian oocyte-stage distribution and impaired oocyte maturation	Ovarian oxidative stress, increased TUNEL-positive cells, altered estradiol/testosterone balance and steroidogenic-enzyme activity, and dysregulation of SIRT1/p53-associated apoptotic, steroidogenic and HPG-axis-related gene expression	[10]
Pristine/contaminated polyethylene microplastics	Fluorescent PE microspheres, 20–27 µm, density 1.005 g/cm^3^	AB-strain zebrafish, Danio rerio, both sexes; exposed from larvae to adults; F1 larvae assessed	1% *w*/*w* PE-MPs in diet; MP-BaP contained 2.68 ± 0.13 µg BaP/g MPs	From 5 dpf through adulthood; reproduction assessed at 3–4 months and F1 development at 6 dpf	MP-BaP reduced relative fecundity and altered egg morphology and yolk area, while sperm quality, embryo viability and hatching were unaffected. Both MP and MP-BaP impaired growth and skeletal development and reduced offspring operculum growth	BaP transport, altered xenobiotic-metabolism and oxidative-stress pathways, including nr1i2, cyp1a, cat, gpx1a, sod1/2 and hsp70/90	[92]
Polystyrene nanoplastics + triclosan	Spherical PS-NPs; nominal size 50 nm, hydrodynamic diameter 54.7 nm, zeta potential −38.3 mV; TCS altered particle dispersion characteristics	Four-month-old adult zebrafish, Danio rerio, AB strain; males and females; F1 embryos assessed	PS-NPs: 1 mg/L; TCS: 0.482–48.2 µg/L in females and 0.361–36.1 µg/L in males	21 days	Co-exposure attenuated TCS-induced ovarian and hormonal disturbances in females but increased testicular TCS accumulation, spermatogenic suppression and hormonal disruption in males; high-dose co-exposure increased F1 embryonic mortality and larval malformations	Sex-specific TCS biodistribution, gonadal histopathology, steroid-hormone disruption and HPGL-axis gene dysregulation; aqp12–dctn2 pathway in females and pck2–katnal1 pathway in males	[93]
Polyethylene microplastics + BPA	Irregular PE-MPs, mean diameter 20 µm; surfaces ranged from smooth to rough and porous; BPA, endocrine-disrupting chemical	Adult zebrafish, Danio rerio, males and females; MLTC-1 Leydig cells also assessed	Zebrafish: PE-MPs 1 mg/L + BPA 1.5 µg/L; cells: PE-MPs 100 µg/mL + BPA 100–150 µM	Zebrafish: 28 days; cells: 48 h	Co-exposure increased GSI in both sexes and altered sex-specific HPG-axis and gonadal steroidogenic gene expression; in MLTC-1 cells, it reduced viability and increased apoptosis and G2/M arrest	HPG-axis disruption; altered Gnrh2/3, Esr1, Ar, Star, Cyp11a1, Cyp19a1a and hydroxysteroid-dehydrogenase genes; apoptosis, cell-cycle disruption and altered steroidogenesis	[7]
Perfluorobutane sulfonate/perfluorobutane sulfonic acid, PFBS	Nonafluorobutane-1-sulfonic acid; 97% purity; molecular weight 300.1 g/mol;	Wild-type adult zebrafish, Danio rerio, males and females	0.14, 1.4 and 14 µM PFBS	28 days	PFBS was detected in the testes and ovaries at 14 µM. Exposure reduced embryo production, hatching rate and GSI in both sexes; the highest concentration reduced mature spermatogenic cells and early- and late-vitellogenic oocytes	Reduced SOD, CAT, GSH, GST and GPx activities; increased MDA and NO; altered estradiol, testosterone and HPGL-axis genes, including cyp19b, esr2b, fshb, lhb, fshr, hsd17β, lhr, cyp19a, vtg1 and vtg2	[76]
Perfluorooctane sulfonate, PFOS, and PFBS	PFOS and PFBS; plastic-particle descriptors: NA	PFOS potassium salt and PFBS tetrabutylammonium salt; purity > 98%	0.2 and 2 µg/L of PFOS or PFBS	2 hpf to 28 dpf; depuration thereafter; adult endpoints assessed at >3 months	Developmental PFOS exposure reduced adult egg production, whereas high PFBS exposure reduced spawning success. Both compounds caused persistent sex-specific changes in growth, organ indices and anxiety-like behavior	Sex-specific hepatic lipidomic disruption involving fatty-acid, sterol/steroid, phospholipid and sphingolipid pathways, with lipid changes associated with reproductive and behavioral endpoints	[13]
Bisphenol A, BPA	BPA, purity > 99%; CAS 80-05-7	Wild-type AB zebrafish, *Danio rerio*; F1 and F2 males, females and offspring assessed	1 nM BPA, nominally 0.228 µg/L; measured concentration 0.372 µg/L	Continuous exposure from 8 hpf to 150 dpf over one or two generations	Female-biased sex ratios in F1 and F2; reduced sperm density, motility, velocity and ATP, with increased lipid peroxidation. Offspring from exposed F2 parents showed delayed hatching and increased malformation and mortality, predominantly through paternal exposure	Altered mitochondrial biogenesis and oxidative phosphorylation, dysregulated canonical and non-canonical Wnt signaling in F2 testes, and reduced dnmt1, dnmt3, dnmt5 and sp3 expression in F2-derived larvae	[65]
Mono-(2-ethylhexyl) phthalate, MEHP	Major DEHP metabolite; CAS 4376-20-9	Adult AB-strain zebrafish, *Danio rerio*, 4–6 months old; males and females	2, 10 and 50 µg/mL MEHP	21 days; semi-static exposure	At 50 µg/mL, MEHP significantly reduced the number of ovulated eggs and inhibited spawning. GSI and gonadal histology were not significantly altered, and the principal reproductive effects occurred in females	Altered female steroid profiles, including increased estradiol, progesterone and cortisol and an increased T/E2 ratio; reduced hepatic VTG and ERα mRNA at 50 µg/mL; ERβ mRNA unchanged; no significant endocrine effects in males	[6]

Abbreviations: ATP, adenosine triphosphate; BPA, bisphenol A; CAT, catalase; DEHP, di(2-ethylhexyl) phthalate; dpf, days post-fertilization; E2, 17β-estradiol; ERα/ERβ, estrogen receptor alpha/beta; F1/F2, first/second filial generation; GPx, glutathione peroxidase; GSH, reduced glutathione; GSI, gonadosomatic index; GST, glutathione S-transferase; HPG axis, hypothalamic–pituitary–gonadal axis; HPGL axis, hypothalamic–pituitary–gonadal–liver axis; hpf, hours post-fertilization; MDA, malondialdehyde; MEHP, mono-(2-ethylhexyl) phthalate; MEHHP, mono-(2-ethyl-5-hydroxyhexyl) phthalate; MPs, microplastics; mRNA, messenger RNA; NA, not applicable; NO, nitric oxide; NPs, nanoplastics; NR, not reported; PE-MPs, polyethylene microplastics; PFAS, per- and polyfluoroalkyl substances; PFBS, perfluorobutane sulfonic acid/perfluorobutane sulfonate; PFOS, perfluorooctane sulfonic acid/perfluorooctane sulfonate; PS-MPs, polystyrene microplastics; PS-NPs, polystyrene nanoplastics; ROS, reactive oxygen species; SIRT1, sirtuin 1; SOD, superoxide dismutase; T, testosterone; TCS, triclosan; VTG, vitellogenin.

**Table 4:** Mixture toxicity and combined exposure outcomes in aquatic organisms.

Mixture/Co-Exposure	Material/Particle Characteristics	Species/Model	Main Reported Reproductive/Developmental Effects	Interaction Pattern	Relevance to Reproductive Toxicity	Ref.
Polystyrene nanoplastics + triclosan	PS-NPs, 50 nm; polymer: polystyrene; pristine commercial particles; shape and surface charge NR; TCS co-exposure altered PS-NP physical characteristics	Adult zebrafish (*Danio rerio*), males and females; offspring endpoints assessed	PS-NPs modified TCS biodistribution. In males, co-exposure increased TCS accumulation in testes and liver, worsened spermatogenesis suppression, and increased embryonic mortality and larval malformations. In females, PS-NPs partly mitigated TCS-induced ovarian inhibition.	Sex-specific interaction; aggravating in males and partly mitigating in females	Shows that nanoplastics can modify contaminant distribution and intensify male reproductive and offspring toxicity	[93]
PFAS + microplastics	PET microplastics with PFAS mixture; PFAS included PFOA and PFOS; particle size, shape, surface charge and weathering state NR	*Daphnia magna*; developmental and reproductive/life-history endpoints	Combined exposure caused developmental problems, delayed sexual maturity, reduced growth and lower reproductive output. Combined effects were reported as approximately 59% additive and 41% synergistic.	Additive and synergistic	Demonstrates that persistent chemicals and microplastics can jointly reduce fitness-related traits and reproductive capacity in aquatic invertebrates	[103]
Polyethylene microplastics + bisphenol A	PE-MPs; polymer: polyethylene; particle size, shape, surface charge and weathering state NR; BPA co-exposure	Zebrafish and MLTC-1 cells	Co-exposure produced stronger endocrine disruption and cellular toxicity than individual exposure, including stronger disruption of HPG-axis-related genes such as *gnrh3*, *esr1* and *ar*.	Synergistic endocrine disruption	Indicates that microplastics can intensify BPA-related reproductive endocrine toxicity	[7]
Polyethylene microplastics + BPA/BPS	PE-MPs, 25 µm; polymer: polyethylene; shape, surface charge and weathering state NR; co-exposure with BPA or BPS	Adult zebrafish parental generation and F1 offspring; adult males and females assessed	Co-exposure aggravated reproductive toxicity in adult zebrafish. Transcriptomic and metabolomic changes involved apoptosis, calcium signaling and glycerophospholipid metabolism. Offspring effects included altered lipid and carbohydrate metabolism.	Additive/interactive parental and offspring effects	Links parental mixture exposure with reproductive impairment and offspring metabolic disruption	[104]
Microplastics + PFAS mixtures	Review-level synthesis of micro/nanoplastics and PFAS; polymer type, particle size, shape, surface charge and weathering state varied among studies	Aquatic food-web/risk synthesis; no single experimental model	The microplastic–PFAS nexus is associated with co-occurrence, adsorption/interfacial interactions, altered transport, trophic transfer, modified uptake/bioaccumulation and enhanced toxicity risk.	Context-dependent mixture risk	Supports mixture-based reproductive risk assessment rather than single-contaminant testing	[105]

Abbreviations: BPA, bisphenol A; BPS, bisphenol S; HPG axis, hypothalamic–pituitary–gonadal axis; NA, not applicable; NR, not reported in the original article or not available from accessible article information; PET, polyethylene terephthalate; PE-MPs, polyethylene microplastics; PFOA, perfluorooctanoic acid; PFOS, perfluorooctane sulfonate; PFAS, per- and polyfluoroalkyl substances; PS-NPs, polystyrene nanoplastics; TCS, triclosan.

**Table 5:** Major ecological and aquaculture impacts of emerging contaminants in aquatic organisms.

Contaminant Group	Representative Species	Key Reproductive Effects	Major Ecological Effects	Aquaculture Impacts
Microplastics/Nanoplastics [121]	Fish, mollusks, crustaceans	Reduced fecundity, impaired gamete quality, decreased larval survival	Food-web disruption and reduced population recruitment	Reduced reproductive performance and offspring quality
PFAS [122]	Fish (e.g., zebrafish, medaka, salmonids)	Endocrine disruption, reduced egg production, impaired embryo development	Bioaccumulation and trophic transfer	Reduced hatching success and stock sustainability
Endocrine-disrupting chemicals (EDCs) [123]	Fish and aquatic invertebrates	Altered sex differentiation, hormonal imbalance, reproductive impairment	Population instability and reduced reproductive fitness	Reduced breeding efficiency and juvenile survival

**Table 6:** Comparison of major treatment and management approaches for reducing emerging contaminants in aquatic systems.

Approach	Main Target Contaminants	Advantages	Limitations	Typical Removal Efficiency *
Membrane filtration [141]	Microplastics, PFAS, EDCs	High removal efficiency; effective for both particles and dissolved contaminants	High cost, membrane fouling, concentrated waste streams	>90–99%
Adsorption (e.g., activated carbon) [142]	PFAS, EDCs	Widely used; effective for many organic contaminants	Reduced effectiveness for some short-chain PFAS; media regeneration required	70–95%
Coagulation–flocculation [143]	Microplastics, EDCs	Cost-effective and suitable for large-scale treatment	Performance depends on water chemistry and particle properties	60–90%
Advanced oxidation/electrochemical treatment [144]	PFAS, EDCs	Can degrade contaminants rather than only remove them	High energy demand and operational costs	60–95%
Constructed wetlands and bio-based treatment [145]	Microplastics, EDCs, selected PFAS	Environmentally friendly; useful for runoff and wastewater management	Variable performance and land requirements	40–80%

* Typical removal efficiencies are approximate values reported in the literature and may vary depending on contaminant properties, treatment conditions, water matrix, and system design.

## Data Availability

No new data were created or analyzed in this study. Data sharing is not applicable to this article.

## Abbreviations

ATP	adenosine triphosphate
BPA	bisphenol A
BPS	bisphenol S
CAT	catalase
DNA	deoxyribonucleic acid
dpf	days post-fertilization
EDCs	endocrine-disrupting chemicals
EPA	United States Environmental Protection Agency
F0	parental generation
F1	first filial generation
F2	second filial generation
F3	third filial generation
GPx	glutathione peroxidase
GSH	reduced glutathione
GSI	gonadosomatic index
GST	glutathione S-transferase
HPG axis	hypothalamic–pituitary–gonadal axis
HPGL axis	hypothalamic–pituitary–gonadal–liver axis
hpf	hours post-fertilization
LC–MS/MS	liquid chromatography–tandem mass spectrometry
MDA	malondialdehyde
MEHP	mono-(2-ethylhexyl) phthalate
MPs	microplastics
mRNA	messenger RNA
NPs	nanoplastics
OECD	Organisation for Economic Co-operation and Development
PE-MPs	polyethylene microplastics
PFAS	per- and polyfluoroalkyl substances
PFBS	perfluorobutane sulfonate
PFOA	perfluorooctanoic acid
PFOS	perfluorooctane sulfonate
PS-MPs	polystyrene microplastics
ROS	reactive oxygen species
SIRT1	sirtuin 1
SOD	superoxide dismutase
WWTP	wastewater treatment plant
